# Geographic and Demographic Differences in the Proportion of Individuals Living in Households With a Firearm, 1990-2018

**DOI:** 10.1001/jamanetworkopen.2024.0562

**Published:** 2024-02-28

**Authors:** Andrew R. Morral, Rosanna Smart, Terry L. Schell, Brian Vegetabile, Emma Thomas

**Affiliations:** 1 RAND Corporation, Arlington, Virginia; 2 RAND Corporation, Santa Monica, California; 3 Contributions completed while at RAND Corporation

## Abstract

**Importance:**

Measures of the proportion of individuals living in households with a firearm (HFR), over time, across states, and by demographic groups are needed to evaluate disparities in firearm violence and the effects of firearm policies.

**Objective:**

To estimate HFR across states, years, and demographic groups in the US.

**Design, Setting, and Participants:**

In this survey study, substate HFR totals from 1990 to 2018 were estimated using bayesian multilevel regression with poststratification to analyze survey data on HFR from the Behavioral Risk Factor Surveillance System and the General Social Survey. HFR was estimated for 16 substate demographic groups defined by gender, race, marital status, and urbanicity in each state and year.

**Exposures:**

Survey responses indicating household firearm ownership were analyzed and compared with a common proxy for firearm ownership, the fraction of suicides completed with a firearm (FSS).

**Main Outcome and Measure:**

HFR, FSS, and their correlations and differences.

**Results:**

Among US adults in 2018, HFR was significantly higher among married, nonurban, non-Hispanic White and American Indian male individuals (65.0%; 95% credible interval [CI], 61.5%-68.7%) compared with their unmarried, urban, female counterparts from other racial and ethnic groups (7.3%; 95% CIs, 6.0%-9.2%). Marginal HFR rates for larger demographic groups also revealed important differences, with racial minority groups and urban dwellers having less than half the HFR of either White and American Indian (39.5%; 95% CI, 37.4%-42.9% vs 17.2%; 95% CI, 15.5%-19.9%) or nonurban populations (46.0%; 95% CI, 43.8%-49.5% vs 23.1%; 95% CI, 21.3%-26.2%). Population growth among groups less likely to own firearms, rather than changes in ownership within demographic groups, explains 30% of the 7 percentage point decline in HFR nationally from 1990 to 2018. Comparing HFR estimates with FSS revealed the expected high overall correlation across states (r = 0.84), but scaled FSS differed from HFR by as many as 20 percentage points for some states and demographic groups.

**Conclusions and Relevance:**

This survey study of HFR providing detailed, publicly available HFR estimates highlights key disparities among individuals in households with firearms across states and demographic groups; it also identifies potential biases in the use of FSS as a proxy for firearm ownership rates. These findings are essential for researchers, policymakers, and public health experts looking to address geographic and demographic disparities in firearm violence.

## Key Points

**Question:**

What is the distribution of individuals living in households with a firearm in the US across years, states, and demographic subgroups from 1990 to 2018?

**Findings:**

In this survey study, estimates of the proportion of individuals living in households with a firearm (HFR) modeled from household survey responses reveal large state and demographic differences in HFR. An estimated 30% of the decline in HFR over the period was associated with growth in demographic groups with low HRF.

**Meaning:**

These results suggest that differences in subgroup exposure to firearms violence and the effects of firearm policies may be sensitive to the large differences in HFR across the population.

## Teaser

This survey study estimates the proportion of US individuals living in households with a firearm by state from 1990 to 2018.

## Introduction

In 2021, nearly 49 000 people in the US died as a result of firearms injuries.^1^ The burden of these deaths is unequally distributed across the country; state firearm death rates differ by an order of magnitude (32.6 per 100 000 in Mississippi vs 3.5 per 100 000 in Massachusetts).^1^ Large differences in violent death rates were also found among demographic groups, although the magnitude of these differences varied across states. For example, Black residents in Wisconsin die in firearm homicides at rates more than 43 times greater than White residents, but firearm homicide rates for Black residents in New Mexico are less than 3-times greater than those for White residents.^1^

Many factors contribute to these large regional and demographic differences in violent death rates,^2,3,4,5,6^ but differences in the rate of firearm ownership may play a role. Firearm acquisition has been shown to be associated with subsequent suicide by any method,^7^ and geographic areas with higher firearm ownership rates have higher total and firearm suicide rates.^8,9^ Similarly, state-level firearm ownership rates are associated with total and firearm homicide rates,^10^ and cohabiting with a handgun owner is associated with elevated risk of death by homicide.^11^

Our understanding of the relationship between firearm ownership and violence has been hindered by the absence of comprehensive, reliable, and systematic data on firearm ownership at the subnational level and over time. While national surveys consistently show that firearm owners are disproportionately men, White, veterans, from nonurban areas, and from southern or East South Central states,^12,13,14^ little is currently known about how ownership rates for these groups vary over time, by state, or across more detailed demographic categories. In fact, the only national survey large enough to provide reliable substate and small-subgroup estimates of firearm ownership, the Behavioral Risk Factor Surveillance System (BRFSS), inquired about household firearm ownership in just 3 survey years approximately 2 decades ago.^15^

In the absence of large and ongoing surveys of firearm ownership, most recent studies that incorporate estimates of state- or substate-level firearm ownership rely on proxy measures that have notable limitations. The standard proxy, the proportion of suicides committed with firearm (FSS), is associated with survey measures of firearm ownership at the state level,^15,16,17,18^ but evidence is more mixed for its association with survey measures of state-level firearm ownership rates over time.^19,20,21^ This makes FSS a problematic proxy for evaluating changes in state-level firearm ownership; this limitation is likely exacerbated for analyses of smaller demographic groups, where comparatively low numbers of suicides result in noisy FSS rates^22^ that typically demonstrate uncertain associations with survey measures of ownership even in cross-sectional analyses.^23^ Prior work has also noted that this proxy may systematically underestimate firearm ownership levels in areas with high long gun rather than handgun ownership, given the predominance of handgun use in firearm suicides.^18^

In this study, we address the need for improved estimates of the proportion of individuals living in households with a firearm, or household firearm ownership rates (HFR), by developing granular, longitudinal estimates of ownership from 1990 to 2018 across states and demographic subgroups defined by gender, race and ethnicity, marital status, urbanicity, and state. Using survey responses on HFR from 16 waves of the General Social Survey (GSS), 1990 to 2018, and 3 waves of the BRFSS, 2001 to 2004, we estimate HFR with multilevel regression and poststratification (MRP) using a bayesian machine learning model.^24^ This approach regularizes year-to-year variability in strata-specific HFR estimates, thereby reducing sampling variance found in the unadjusted survey estimates. The model produces HFR estimates for each of 744 analytic strata (defined by state and demographic subgroups) in each year, which can be aggregated using poststratification weights to be representative of larger population groups. We describe these estimates and then compare our survey-based HFR estimates with FSS as measures of firearm ownership over time and across states and demographic subgroups. All estimates are publicly available and can be visualized or downloaded online.^25^

## Methods

This survey study was approved by the RAND Corporation’s institutional review board. Informed consent was waived because all data were deidentified before they were received. It follows the American Association for Public Opinion Research (AAPOR) reporting guideline.

### Data Sources

Self-reports of firearms in the household among adults 18 years and over were drawn from 3 administrations of the BRFSS (192 700 individuals in 2001, 221 920 in 2002, and 276 408 in 2004), a large, random-digit dialed telephone survey conducted annually by the US Centers for Disease Control and Prevention (CDC), and 16 administrations of the GSS, a smaller in-person survey of adults sampled within households from 1990 to 2018 (22 430 individuals). Self-reported sex, race and ethnicity, marital status, and county urbanicity were selected as the primary demographic characteristics on which to stratify population groups because of large disparities in firearm violence risk each are associated with.^26^

Population estimates for adults in each state, year, and demographic stratum were constructed from the National Cancer Institute’s Surveillance, Epidemiology, and End Results Program (SEER) models^27^ and the US Census Bureau’s American Community Survey (ACS).^28^

Suicide and firearm suicide rates for adults 18 and over by state, year and demographic strata were calculated from the CDC restricted multiple cause of death data set for states and all counties. Additional details on these data sources and data preparation are found in eAppendix 1 in Supplement 1.

### Statistical Analysis

We used MRP to estimate strata-specific HFR.^29^ MRP uses partial pooling to improve strata-specific estimates; HFR information from individuals who share demographic characteristics is used to improve strata-specific HFR estimates, even for strata with sparse data. These regularized strata-specific estimates are then aggregated into population-level HFR prevalence estimates using poststratification weights corresponding to the population size of each stratum. MRP has been shown to produce reliable subnational estimates from surveys designed only for national estimates.^30^

Consistent with current recommendations^24^ we used a flexible machine learning algorithm, Bayesian Additive Regression Trees^31^ (BART), to predict self-reported HFR as a function of the following variables: year, state, gender (man vs woman), marital status (married vs not married), race and ethnicity (White non-Hispanic or American Indian or Alaskan Native vs all other; White and American Indian vs other racial and ethnic groups, hereafter), urbanicity (large densely populated county vs all other counties), a binary indicator for survey (GSS vs BRFSS), and a smoothed annual estimate of the proportion of suicides committed with a firearm (FSS) for each analytic stratum, with strata defined by the intersection of state, gender, marital status, race, and urbanicity. Further details on categorization of race and ethnicity are available in eAppendix 1 in Supplement 1. This model was used to produce a posterior distribution of the predicted probabilities of living in a household with a firearm within each of the 744 analytic strata in each year. These estimates for each stratum and state are aggregated to construct population HFR estimates using poststratification weights proportional to the size of each stratum in the target population.

The predictive model used a smoothed FSS estimate, rather than FSS directly calculated from suicide rates, because a large fraction of year, state, and strata populations had no suicides or very few, meaning FSS was undefined or measured with great variability.^32^ Instead, we used a separate BART model to estimate the FSS as a function of year, state, and demographic strata. Smoothed FSS was then calculated as the model-predicted probability of a firearm suicide for each stratum, state, and year. Smoothed FSS is used as a predictor in the firearm ownership model; for analyses looking at the similarities and differences between HFR and FSS, we use the standard measures of FSS as these can be calculated for the larger populations used in the comparisons.

Additional analytic details and discussion are found in eAppendix 2 in Supplement 1. Estimates of HFR, HFR without smoothed FSS as a predictor, and smoothed FSS are available online.^25^

## Results

**Figure 1.**
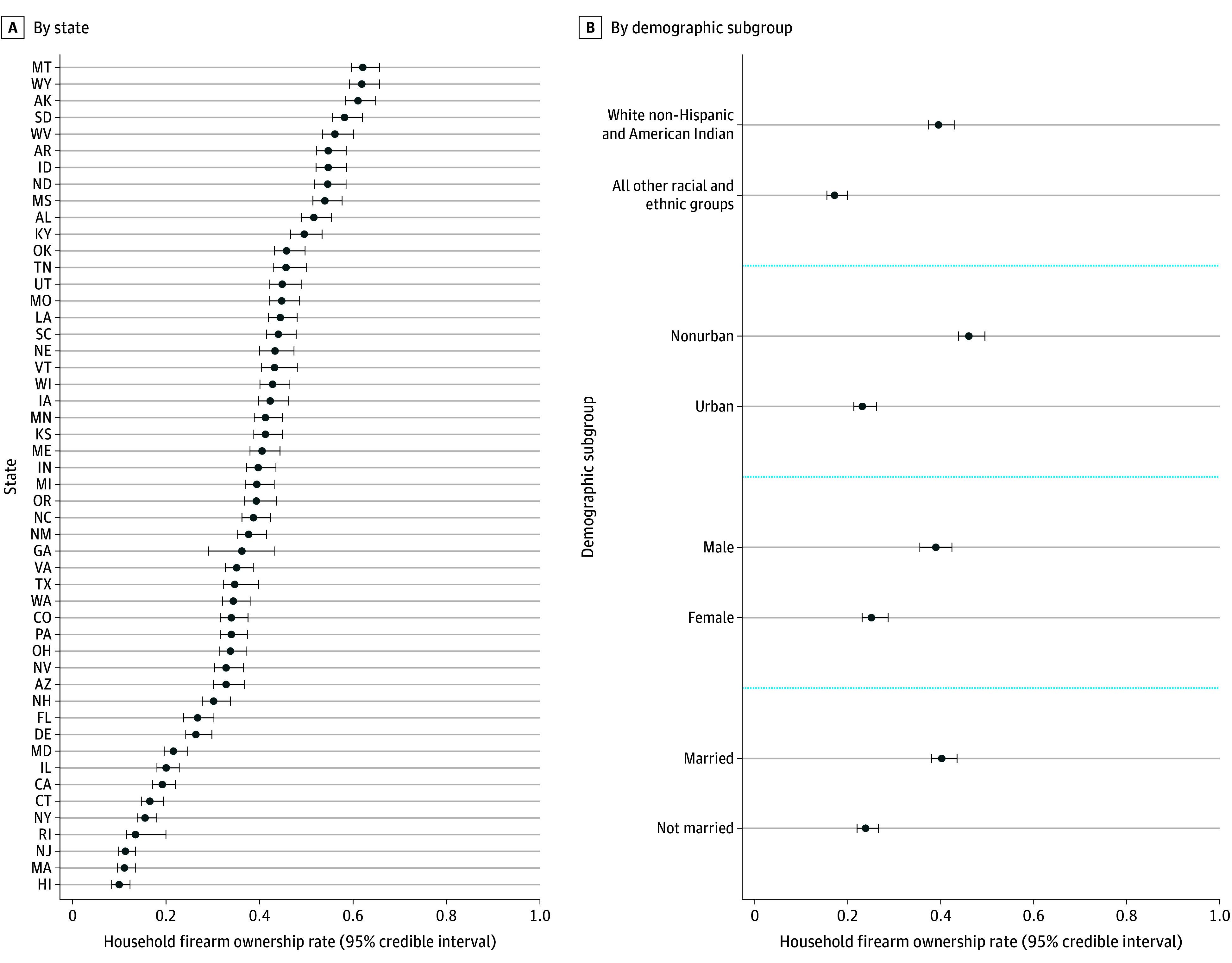
Estimates of Proportion of Individuals Living in Households With Firearms (HFR) in 2018, by State and Demographic Subgroups

A total of 713 458 survey respondents were available for these analyses. HFR varied substantially across the geographic and demographic factors studied. Across states in 2018, poststratified population estimates of HFR ranged from a low of 9.9% (95% credible interval [CI], 8.4%-12.3%) in Hawaii to a high of 62.1% (95% CI, 59.6%-65.7%) in Montana (Figure 1). The marginal differences in HFR across the demographic factors were also substantial. In 2018, White and American Indian populations had HFR (39.5%; 95% CI, 37.4%-42.9%) more than twice as high as HFR for other racial and ethnic groups (17.2%; 95% CI, 15.5%-19.9%); those in nonurban counties (46.0%; 95% CI, 43.8%-49.5%) had twice the HFR as urbanites (23.1%; 95% CI, 21.3%-26.2%); men (38.9%; 95% CI, 35.5%-42.4%) had higher HFR than women (25.1%; 95% CI, 23.1%-28.7%); and married populations had higher HFR (40.2%; 95% CI, 38.0%-43.5%) than the unmarried (23.8%; 95% CI, 22.0%-26.6%). Combining these effects produces differences that are quite large. In 2018, the highest rates of HFR were among married, nonurban, White and American Indian men (65.0%; 95% CI, 61.5%-68.7%), almost an order of magnitude higher than the estimated HFR for unmarried, urban women from other racial and ethnic groups (7.3% in 2018; 95% CI, 6.0%-9.2%).

At the national level, the estimated prevalence of HFR has declined by 7 percentage points over the studied period, from 39% (95% CI, 37%-42%) in 1990 to 32% (95% CI, 30%-35%) in 2018 (eFigure 1 in Supplement 1). Declining HFR was concentrated in the late 1990s, followed by a period of relatively stable ownership. More recently, from 2014 to 2018, there has been a small increase in HFR.

The machine learning model that underlies these estimates allows for complex interactions between temporal, geographic, and demographic characteristics. However, the simple main effects of geographic and demographic features capture most of the variation in strata-level estimates. Specifically, 89% of the variability in strata-level HFR estimates was explained by the main effects of time, state, and demographics (race and ethnicity, gender, urbanicity, and marital status). The 50 state effects contributed the most to explaining variation (33%), followed by race and ethnicity (19%), gender (19%), marital status (15%), urbanicity (13%), and year (2%). Interactions between these factors matter far less in explaining the HFR estimates. The most important interaction is marital status with gender (married women are far more likely to live in a home with a gun than unmarried women); adding this interaction to the 6 main effects explains 93% of the variance in HFR, 3% more than the main effects alone.

The relatively small contribution of time (2%) implies that HFR for individual strata shows little variability over time relative to other studied factors. However, even if HFR rates among demographic subgroups remained relatively stable, changes in the population composition of states and the nation resulted in larger shifts in population HFR over the study period. For instance, the proportion of the population who are unmarried, urban, and from minority racial or ethnic groups has increased over the past 2 decades,^33,34^ and these groups have lower HFR.

**Figure 2.**
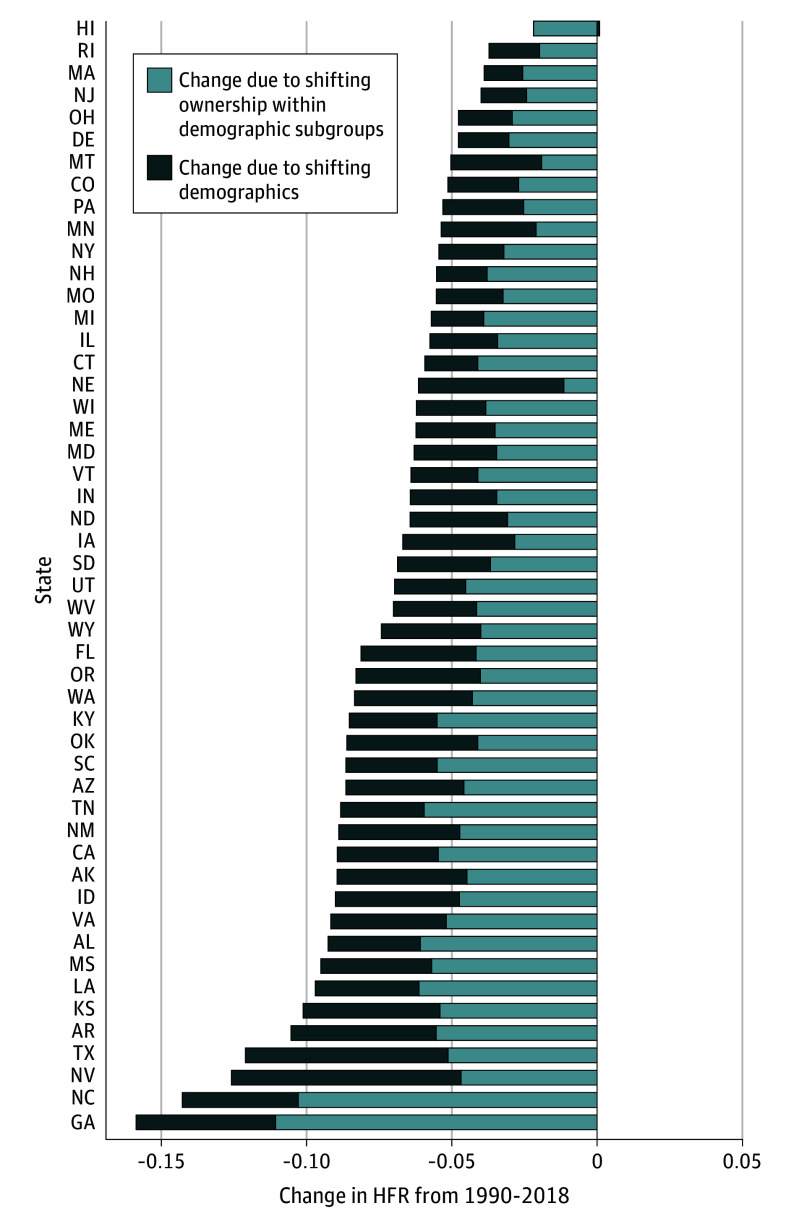
Change in Proportion of Individuals Living in Households With Firearms (HFR) Between 1990 and 2018, by State, Decomposed by Source of Change

To better understand this variation over time, we decompose each state’s estimated change in HFR between 1990 and 2018 into 2 components: (1) changes in HFR within demographic strata (ie, the change in expected HFR if population composition were held constant at 1990 values) and (2) changes in HFR driven by changes in states’ demographic composition. Changing demographics explain more than 30% of the decline in HFR for all states, and in 4 states (Minnesota, Montana, Nevada, and Nebraska) changing demographics explain more than 60% of the decline (Figure 2). Differences in demographic composition across states explain an even larger portion of the difference between state and national HFR rates: on average across states in 2018, 43% of this difference is explained by demographic differences between states and the US as a whole (eAppendix 3 and eFigure 3 in Supplement 1).

### Comparisons With FSS

**Figure 3.**
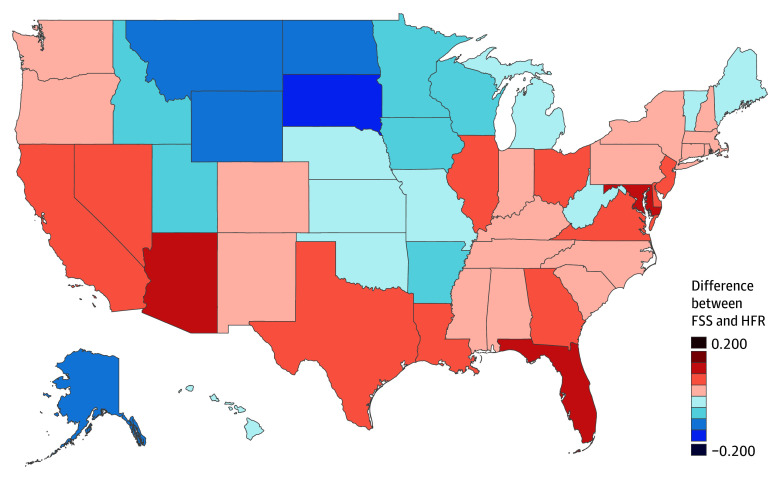
Differences Between Firearm Ownership Rates as Proxied for by FSS and Survey-Based HFR Estimates, Averaged Over 1990 to 2018 FSS indicates proportion of suicides committed with firearm; HFR, proportion of individuals living in households with firearms.

Across states and years, the correlation between HFR and FSS was r = 0.84. Although this overall correlation is high, there is evidence that the errors are not random and that FSS is a biased proxy of HFR for some states. We investigated this by creating an FSS proxy for the HFR measure, which is the linear transformation of FSS that has the smallest squared discrepancy with HFR over the 1450 state-years in the data. Figure 3 shows the differences between the FSS proxy and HFR for each state, averaged over time. Reliance on the FSS proxy underestimates HFR for some states (eg, North Dakota, Minnesota, and Wyoming) by nearly 20 percentage points and overestimates HFR for others (eg, California, Florida, and Maryland) by over 10 percentage points (eFigure 4 in Supplement 1).

Using analogous methods, we find that the FSS proxy systematically under- or overestimates survey measures of HFR across demographic strata, when averaged over states and across time. It overestimates HFR among populations from other racial groups by an average of 12 percentage points, urban HFR by 9 percentage points, and HFR among the unmarried by 11 percentage points. It underestimates HFR among women by 6 percentage points. Greater disparities between the FSS proxy and HFR were found for more granular subgroups, many of which displayed marked time trends indicating that the association between FSS and HFR has changed systematically for some groups (eFigure 5 and eFigure 6 in Supplement 1).

## Discussion

Understanding the landscape of firearm ownership in the US and how it varies across time, space, and demographics is critical for answering basic questions about the risks and benefits of keeping a firearm in the home and where or for whom firearms are more or less available.^15^ Prior studies seeking to account for HFR have been constrained in the granularity with which reliable estimates can be produced, and often produce these estimates only for a short period in the early 1990s when the BRFSS survey collected information on household firearms.^14,15^ Using MRP to produce subnational estimates from nationally representative surveys, this study provides the first detailed, longitudinal estimates of HFR by state and demographic subgroups. We found striking variability in this measure across demographic groups at the national and substate levels, and evidence that differences in the demographic composition of states and the nation over time explain a substantial portion of the observed heterogeneity in HFR rates.

Where our estimates can be compared with others, they were similar when the data used was the same BRFSS or GSS data used in our own models.^14,35^ More compelling evidence of validity comes from several recent state-specific BRFSS surveys that were not included in our analyses. Our HFR estimates are generally within a few percentage points of the published estimates for the 7 states that collected this more recent firearm data^36,37,38^ (eAppendix 3 in Supplement 1).

The HFR estimates produced in this study reveal potentially important dynamics in exposure to household firearms and their associated risks. For instance, our results showed a 250% increase in HFR among married women compared with unmarried women (from 15% to 37%), a far greater increase than that found between unmarried and married men (35% and 45%, respectively). As such, marriage is disproportionately associated with exposure to household firearms for women compared with men.

Despite stable subgroup HFR rates, all states and the nation experienced declines in HFR over the study period. We found that 30% of the reduction in state HFR rates is associated with changes in the demographic composition of state populations over time. That is, groups with higher HFR have been declining as a proportion of state populations, resulting in overall reductions in HFR. This finding highlights a previously unrecognized danger of continuing to rely on HFR estimates from BRFSS surveys conducted more than 20 years ago: studies using these old HFR estimates are likely to overestimate current HFR, and disproportionately so for states with the largest demographic changes.

This study contributes to recent efforts to understand the validity of using FSS as a proxy for HFR at the state level^15,16,17,18,19,20,23^ or across racial groups.^35^ Our HFR estimates have a cross-state correlation with FSS of r = 0.84, which is comparable to correlations found between state FSS and other survey measures of HFR.^19,39^ A proxy with 70% of its variance associated with the desired construct would be very good if what remained was random error. However, we found that differences between the FSS proxy and our direct measures of HFR are not random but represent systematic biases. The FSS proxy overestimates HFR for minority racial and ethnic groups and unmarried populations by more than 10 percentage points. State-level estimates of HFR using the FSS proxy are 10 percentage points too high for some states, and 20 percentage points too low for others.

These results suggest that preferences for specific suicide methods vary considerably across geographic and demographic groups and over time in ways that do not align with differences in self-reported HFR. Therefore, FSS is not just a proxy for firearm ownership but also for gender, race, geography, and time. Because of this, researchers should not interpret associations with FSS as equivalent to associations with gun ownership. FSS is associated with gun ownership, so may still be a useful control covariate in research where: it is not interpreted as equivalent to gun ownership; where factors such a race, gender, geography, and time are also included as covariates; and where the outcome being predicted is not itself a subset of the suicides used to compute FSS.

### Limitations

This study had several limitations. As with all recent work on HFR, we treat survey-based self-reports of household firearms as the standard against which to evaluate FSS. Survey measures have been found to have the highest correlation with latent household firearm constructs compared with other candidate measures.^15^ Moreover, the validity of the FSS proxy rests entirely on its correlation with survey responses. Where FSS differs from survey-based estimates like ours, there is little justification for suspecting that FSS is more likely to be accurate, even accepting that these survey-based estimates may be subject to a range of biases.^40^ To the extent that preference for firearms as a means of suicide differs across groups or over time, a portion of the variance in FSS is tethered not to HFR but to other factors that influence selection of suicide means.^41^ Nevertheless, HFR is subject to the same sources of bias that survey measures are and those errors are of uncertain direction and magnitude.

Another limitation is that comparatively little survey data was available in non-BRFSS years. Indeed, in some years there were relatively few or no respondents in some state strata. However, this is the problem MRP was designed to address: where less information is available, more pooling of information occurs across similar analytic strata or across adjacent years. The credible intervals convey the uncertainty in the population values given the available data.^29^

## Conclusions

This survey study of HFR provides detailed, publicly available HFR estimates, highlighting key disparities among individuals in households with firearms across states and demographic groups. The study also identifies potential problems in the use of FSS as a proxy for HFR. These findings are essential for researchers, policymakers, and public health experts looking to address geographic and demographic disparities in firearm violence.

## Notes

Supplement 1. **eAppendix 1.** Data and Strata **eAppendix 2.** Methodological Details **eAppendix 3.** Supplementary Analyses **eFigure 1.** Change in Household Firearm Ownership Rate, National (Thick Black Line) and by State, 1990-2018 **eFigure 2.** Estimates of the Proportion of Individuals Living in Households With Firearms for 16 US Demographic Subgroups (With 95% Credible Intervals) **eFigure 3.** Differences Between State and National HFR Rates in 2018: Total Difference (Unadjusted) and After Adjusting for Demographic Differences Between States and the Nation **eFigure 4.** Differences Between Firearm Ownership Rates as Proxied by FSS and Survey-Based HFR Estimates, by State and Year **eFigure 5.** Differences Between Firearm Ownership Rates as Proxied by FSS and Survey-Based HFR Estimates, by Subgroup and Year **eFigure 6.** State Household Firearm Ownership Rates (HFR) and FSS Rates by Demographic Subgroups and Years **eTable.** Comparisons of HFR With State and Substate-Level Estimates From State-Specific BRFSS Module Data Presented in Published Literature

Supplement 2.
Data Sharing Statement
