# Supplementary material for: Geographic and Demographic Differences in the Proportion of Individuals Living in Households With a Firearm, 1990-2018
Source: JAMA Netw Open. 2024 Feb 28;7(2):e240562. doi: 10.1001/jamanetworkopen.2024.0562 (PMC10902733; doi:10.1001/jamanetworkopen.2024.0562)
Supplement: Supplement 1. — eAppendix 1. Data and Strata eAppendix 2. Methodological Details eAppendix 3. Supplementary Analyses eFigure 1. Change in Household Firearm Ownership Rate, National (Thick Black Line) and by State, 1990-2018 eFigure 2. Estimates of the Proportion of Individuals Living in Households With Firearms for 16 US Demographic Subgroups (With 95% Credible Intervals) eFigure 3. Differences Between State and National HFR Rates in 2018: Total Difference (Unadjusted) and After Adjusting for Demographic Differences Between States and the Nation eFigure 4. Differences Between Firearm Ownership Rates as Proxied by FSS and Survey-Based HFR Estimates, by State and Year eFigure 5. Differences Between Firearm Ownership Rates as Proxied by FSS and Survey-Based HFR Estimates, by Subgroup and Year eFigure 6. State Household Firearm Ownership Rates (HFR) and FSS Rates by Demographic Subgroups and Years eTable. Comparisons of HFR With State and Substate-Level Estimates From State-Specific BRFSS Module Data Presented in Published Literature [file jamanetwopen-e240562-s001.pdf]

Supplemental Online Content

Morral AR, Smart R, Schell TL, Vegetabile B, Thomas E. Geographic and demographic differences in the proportion of individuals living in households with a firearm, 1990-2018. *JAMA Netw Open.* 2024;7(2):e240562. doi:10.1001/jamanetworkopen.2024.0562

eAppendix 1. Data and Strata

eAppendix 2. Methodological Details

eAppendix 3. Supplementary Analyses

eFigure 1. Change in Household Firearm Ownership Rate, National (Thick Black Line) and by State, 1990-2018

eFigure 2. Estimates of the Proportion of Individuals Living in Households With Firearms for 16 US Demographic Subgroups (With 95% Credible Intervals)

eFigure 3. Differences Between State and National HFR Rates in 2018: Total Difference (Unadjusted) and After Adjusting for Demographic Differences Between States and the Nation

eFigure 4. Differences Between Firearm Ownership Rates as Proxied by FSS and Survey-Based HFR Estimates, by State and Year

eFigure 5. Differences Between Firearm Ownership Rates as Proxied by FSS and Survey-Based HFR Estimates, by Subgroup and Year

eFigure 6. State Household Firearm Ownership Rates (HFR) and FSS Rates by Demographic Subgroups and Years

eTable. Comparisons of HFR With State and Substate-Level Estimates From State-Specific BRFSS Module Data Presented in Published Literature

This supplemental material has been provided by the authors to give readers additional information about their work.

©2024 Morral AR et al. *JAMA Network Open.*

eAppendix 1. Data and Strata

The data used to estimate firearm ownership were individual-level, self-reported household firearm ownership collected from 16 administrations of the General Social Survey (GSS, n=22,430; [1]) between 1990 and 2018, and three waves of the Behavioral Risk Factor Surveillance System (BRFSS) conducted between 2001 and 2004 (n=691,028).[2] The overall estimation approach is longitudinal multilevel regression and poststratification[3] (MRP) that uses a BART machine learning model[4] as the multilevel regression model. This general approach is similar to that advocated by Bisbee.[5] The model predicts self-reported household firearm ownership as a function of: year, state, gender (male v. female), marital status (married v. not married), race/ethnicity (White non-Hispanic or American Indian or Alaskan Native v. all other), urbanicity (large densely populated county v. all other counties), a dichotomous indicator for survey (GSS v BRFSS), and a smoothed annual estimate of FSS for each analytic stratum (with strata defined by the intersection of state, gender, marital status, race, and urbanicity).

This model was used to produce annual predictions of firearm ownership within each analytic stratum. These stratum-level estimates are then combined to aggregation of interest (e.g., state or women) using poststratification weights that represent the population size of each stratum in each year. Population size estimates were taken primarily from the National Cancer Institute's Surveillance, Epidemiology, and End Results Program models[6] (SEER), with information on marital status estimated using data from the U.S. Census. Each of these steps in the analysis are discussed in more detail below.

Strata Definitions

The goal of the project was to produce accurate firearm ownership estimates at a variety of levels of aggregation in a way that captures much of the demographic and geographic variation in ownership rates. However, the choice of analytic strata was constrained in two ways: (1) we needed to avoid complex demographic variables that would pose estimation problems due to minimal representation in the underlying survey data, and (2) we needed to have strata that had identical definitions across all data sources over time, including: the two surveys (GSS, BRFSS), the National Vital Statistics System, and

©2024 Morral AR et al. *JAMA Network Open.*

population size estimates from the Surveillance, Epidemiology, and End Results Program or the U.S. Census. We settled on strata defined by four dichotomous demographic indicators across the 50 US states.

1. *Urbanicity*. The definition of urbanicity is based on county of residence. It is unique to this study and designed to fit within the privacy limitations of the BRFSS data, which suppresses country indicators for counties with less than 50 respondents. Initial urbanicity designations for all counties were based on 2010 county population and population density estimates from the U.S. Census. Specifically, urban counties are those with a population of 200,000 or more as well as a density of 0.5 or greater, where density is defined as population x 10,000 / non-water-land-area-in-meters-squared. All counties not qualifying as urban were counted as not urban. A small number of counties with between 200,000 and 300,000 residents suppressed county information in BRFSS due to too few respondents. Those counties were reclassified as nonurban for all analyses. Under this definition, 62% percent of the US population in 2018 lived in an urban county, and seven states have no urban subpopulations.
2. *Marital status*. Married vs any other marital status.
3. *Race/ethnicity*. We distinguished two broad groupings of self-reported race among survey respondents. White non Hispanic or American Indian or Alaskan Native (AIAN) vs all other race/ethnic combinations. The combination of AIAN with non Hispanic White is unusual but has several advantages for this project. First, estimating AIAN firearm ownership separately could not be supported in the survey data both because the GSS did not include it as an option in the earliest years of the data and because of the low numbers of AIAN respondents in almost all years and states even when it was asked. Second, AIAN and non Hispanic white are the two race/ethnic groups with above average firearm ownership and so this method of collapsing these complex variables provides a better fit to the data. Third, distinctions between AIAN and non Hispanic White classifications are often error prone in vital statistics data[7] and inconsistent in self reports by the same individual over time.[8]
4. *Gender*. Male vs female as self-reported gender.

©2024 Morral AR et al. *JAMA Network Open.*

The analytic strata are defined by the cross-classification of these demographic variables and state. Because not all states have both urban and nonurban counties, the total number of strata are 744 which is slightly less than the total possible (2 x 2 x 2 x 2 x 50 = 800).

Data sources

Our analyses require information on firearm ownership, the adult population, and suicides within each of 16 subgroups measured in each state and year between 1990 and 2018. In this section we describe how we identified population subgroups in these key data sources, and how we imputed missing or erroneous values in the data.

Self-reported household firearm ownership

We used two survey series to gather information about self-reported household firearm ownership over time: three administrations of the Behavioral Risk Factor Surveillance system (BRFSS) between 2001 and 2004 and 16 administrations of the General Social Survey (GSS) from 1990 to 2018. Together, these sources provided N=713,458 household firearm ownership survey responses.

The Behavioral Risk Factor Surveillance System. BRFSS is an annual random digit dialed telephone survey that collects state-representative self-reports by adults 18 and older on a range of health behaviors. Household firearm ownership questions have only been asked in all states in the three years we include in this study. During these years, BRFSS used post-stratification to weight survey responses to be population representative.

A question about household firearm ownership has been asked nationally in the core module of the BRFSS survey only three times, and we use ownership responses from each of these administrations. In 2001, the question asked was:

> The next question is about firearms, including weapons such as
> pistols, shotguns, and rifles; but not BB guns, starter pistols, or guns

©2024 Morral AR et al. *JAMA Network Open.*

that cannot fire. Are any firearms now kept in or around your home? Include those kept in a garage, outdoor storage area, car, truck, or other motor vehicle. (BRFSS FIREARM3 item.)

For the 2002 and 2004 administrations of this survey, the question was modified but remained substantively similar:

The next questions are about firearms. We are asking these in a health survey because of our interest in firearm-related injuries. Please include weapons such as pistols, shotguns, and rifles; but not BB guns, starter pistols, or guns that cannot fire. Include those kept in a garage, outdoor storage area, or motor vehicle. Are any firearms now kept in or around your home? (BRFSS FIREARM4 item.)

We define household firearm owners as those who indicate there is a firearm in or around their home. We include in this analysis only those respondents who completed the household firearm ownership question who were sampled in one of the 50 states (N = 192,700, 221,920, and 276,408 for the 2001, 2002, and 2004 BRFSS administrations).

Public-use BRFSS data files for the study years include the year of survey administration, the state and county of residence, respondents' gender, and respondent marital status. Two variables, preferred race (_PRACE) and race-ethnicity grouping (RACE2) were used to construct the race variable. Specifically, if RACE2 = 1 (White-only, non-Hispanic) or _PRACE= 5 (American Indian or Alaskan Native), then the respondent was categorized as White non-Hispanic, American Indian or Alaskan Native. All other respondents were coded as in the other category. These included respondents selecting "Asian", "Native Hawaiian or other Pacific Islander", "Other", "Do not know", and "Refused." Therefore, all 16 subgroups were identified among survey respondents in each state and year.

©2024 Morral AR et al. *JAMA Network Open.*

The General Social Survey. The GSS is a nationally representative survey of public opinion, attitudes, and behaviors that uses multistage probability sampling of household units, with information collected in face-to-face interviews and some computer assisted personal interviews conducted by phone. Within households, each adult older than 18 was given equal probabilities of selection into the sample. As such, GSS survey responses can be weighted to be representative of U.S. households or U.S. adults.

Sixteen administrations of the GSS survey were used in this study: 1990 (n=904), 1991 (n=986), 1993 (n=1,065), 1994 (n=1,983), 1996 (n=1,916), 1998 (n=1,868), 2000 (n=912), 2002 (n=921), 2004 (n=891), 2006 (n=1,988), 2008 (n=1,333), 2010 (n=1,264), 2012 (n=1,296), 2014 (n=1,683), 2016 (n=1,868), 2018 (n=1552). Case counts include only those respondents who answered the household firearm ownership question in each survey: "Do you happen to have in your home (IF HOUSE: or garage) any guns or revolvers?" and respondents could respond affirmatively that they have a gun in their home, negatively that they did not have a gun in their home, or they may be coded as having not provided an answer (refusing to answer, responding that they did not know or could not choose, or skipped the question).

The restricted-access data we used provided the survey year, state and county of residence for each respondent, as well as respondents' self-reported marital status. Race information was collected in different ways over the time series. Prior to and including the year 2000, the survey included a three-option race variable (White, Black, Other) and no ethnicity variable. For these years, we count respondents indicating "White" as belonging to the White non-Hispanic race/ethnicity category; all others are coded as not in this race/ethnicity category. After 2000, more race options became available, and a question about Hispanic heritage was added. In these years, we count respondents who indicate they are White (racecen1=1) and not Hispanic (Hispanic =1) as in the White non-Hispanic race/ethnicity category.

Population data

The size of the population for each state, year, and demographic subgroup are required to post-stratify strata-specific estimates of firearm ownership to generate ownership estimates for aggregations of strata, such as for state or national estimates that combine across multiple strata characteristics. With a

©2024 Morral AR et al. *JAMA Network Open.*

few exceptions, these population estimates were derived from the National Cancer Institute's Surveillance, Epidemiology, and End Results Program (SEER) models.[6] SEER estimates are constructed from the US Census' county population estimates by age, gender, race, and Hispanic origin. Specifically, we use the SEER program's bridged-race estimates, which provide a consistent categorization of race/ethnicity for 1990 through 2019, despite changes in question wording used in Census surveys. The four bridged-race categories are: White, Black, Asian and Pacific Islanders, and American Indian and Alaskan natives. The SEER estimates also differentiate race groupings by Hispanic origin. To match the universe of respondents to the GSS and BRFSS surveys, we restricted population estimates from SEER to those aged 18 and over.

SEER provides estimates of population size for every stratum defined by the cross-classification of year, state, county, gender, and race, but does not indicate how many individuals within each of those strata are married. Thus, we use the US Census Bureau's American Community Survey (ACS) to estimate the proportion married in each substate stratum and by year.[9] The methods for this imputation are described in the section "Methodology for imputing marital status" below.

Suicide Incidents

We required suicide data for each year and population stratum for our analyses of the relationship between our HFR estimates and the FSS proxy for ownership (i.e., the proportion of suicides completed with a firearm). The restricted *Multiple cause of death, state and all counties* dataset from the National Vital Statistics System includes information on the year each death occurred, the mechanism of death (e.g., firearm, non-firearm) and the cause of death (e.g., suicide, homicide). Other decedent information includes the state and county where the death occurred and the decedents' gender, age, race, ethnicity, and marital status. Using these data for the years 1990-2018, we calculated FSS for each state and for each demographic subgroup at the national level for use in analyses reported in the body of the paper designed to understand how FSS related to HFR. We also create a model-based estimate of FSS used as a predictor in the main MRP model; those analyses and the rationale for its inclusion as a predictor are discussed in the analysis section below.

©2024 Morral AR et al. *JAMA Network Open.*

Imputation of missing data

Imputing marital status population size estimates. SEER data provide population counts for each of eight subgroups in each state and year defined by the cross-classification of gender, race and urbanicity, but SEER does not include information on marital status. To generate population size estimates for the full set of 16 subgroups (used for poststratifying HFR estimates that aggregate over strata), we need to estimate the fraction of each of the eight SEER subgroups that are married in each state and year. We impute these annual strata-specific marriage rates using data from the American Community Survey from 2006 through 2019 and the decennial censuses conducted in 1990 and 2000.[9] Specifically, we first estimate the fraction of each of the 8 strata that are married from the Census/ACS data, then apply these fractions to the SEER population estimates to divide each into a married and unmarried population.

We did not use 2010 decennial census data because marital status is unavailable in public use samples that include our other strata variables, but it is available in the 2010 ACS. We did not use the 2005 ACS due to changes in the weighting methodology from 2006 onwards that affected marital rate estimates,[10] and because group quarters were excluded in 2005, leading to over-estimation of marital rates.[11]

Year, state, gender, and race have the same definition in the ACS/Census and SEER data. We computed single race estimates for 2016 ACS onwards using the same "race bridge" definition used by SEER. County, which is required to compute our urbanicity variable, is not identified in the public ACS/Census microdata provided by IPUMS that also includes marital status and our other stratifying variables.[9] The finest, fully-identified level of geographic information provided is the Public Use Microdata Area (PUMA). There is no perfect mapping between PUMAs and counties. However, the Missouri Census Data Center provides weighted "crosswalks" between PUMAs and census counties as measured in 1990, 2000 and 2010 decennial counts.[12] The weights provided by these crosswalks were used to approximate the number of respondents in each census/ACS year residing in each county, within population strata.

To estimate strata-specific estimates of marital rates over time, we modeled these rates within strata as a function of time. These models were weighted by the person-level population weights provided

©2024 Morral AR et al. *JAMA Network Open.*

with the IPUMs public use data, with a modification: the weights for each census/ACS year were multiplied by the total number of respondents divided by the sum of the weights. Thus, the sum of the rescaled weights in each census/ACS year was proportional to the number of respondents in each year, giving higher weight to the larger samples when smoothing marital rates over time. Note that as our goal was only to obtain point estimates of marital rates, the impact of the weights on standard error estimates was not a concern.

We smoothed marital rates within strata and over time using natural cubic splines with one internal knot at 2003 and boundary knots at the limits of the data in 1990 and 2019, which fit the data better than a simple linear model. We applied the resulting marriage rates to the eight population subgroup estimates derived from the SEER data, to produce the full set of 16 subgroup-specific population estimates for each state and year.

Imputing county population sizes in Hawaii. SEER does not provide county-level estimates for the state of Hawaii prior to 2000.[6] We therefore imputed the number of people in urban and rural counties in Hawaii for 1990 through 1999 using decennial census and inter-census ACS data within strata defined by year, gender, and race; the number of married people was then imputed using the same methodology as for all states, described in the previous section.

We first created tables of counts of urban and rural individuals in each census/ACS year and county by gender and race. We used the same census/ACS tables as described in the previous section on marital rates, except that we included 2005 ACS data and summed over marital status. We then estimated the fraction of people in urban counties by gender and race, smoothed over years. To achieve smoothing, we used natural cubic splines with one internal knot at 2003 and boundary knots at the limits of the data (1990 and 2019). We included the 2005 onwards ACS datapoint as we would otherwise only have two datapoints for interpolation within each stratum (1990 and 2000 census). However, these datapoints had relatively little impact on the urbanicity estimates prior to 2000. Finally, we applied urban fraction estimates to SEER population counts from 1990 to 1999 by race, gender, and year to estimate the number of people in urban and rural counties in Hawaii.

©2024 Morral AR et al. *JAMA Network Open.*

Imputing urbanicity for Georgia suicides in 2008. Some administrative issues with changing data systems led to missing county of death and marital status in the NVSS file for a large proportion of deaths in Georgia in 2008. In the mortality data we received from NCHS, county of death had been imputed to the largest Georgia county (Fulton county; see Miniño et al., 2011). Whereas county of *occurrence* was often imputed, county of *residence* at the time of death appeared to be available for most of these records. In Georgia, county of occurrence and residence are highly correlated for suicide deaths. For instance, in 2007 and 2009 – years without excessive missing data -- 92% of suicide deaths were correctly categorized as urban/not urban using county of residence rather than county of occurrence.

Therefore, for Georgia in 2008, we used county of *residence* rather than county of *occurrence* to identify urbanicity status for deaths meeting all of the following criteria: (a) missing marital status; (b) county of occurrence listed as Fulton County, Georgia; (c) year of death was 2008. Although some of these counties of residence were outside Georgia, because the death occurred in Georgia our mortality tables assign these deaths to Georgia but use urbanicity status from the state of residence.

Imputing marital status in mortality records. Marital status in the NVSS data was frequently missing for Georgia deaths in 2008 and 2009, for the same administrative reasons that county of occurrence was frequently missing (note that the issue with missing county information resolved for the 2009 data). Mortality data for most other states, years, and population subgroups had no missing marital status or low levels of missingness. However, since we needed to impute unknown marital status for Georgia records, we also imputed it for all other NVSS data using simple linear interpolation. Specifically, we fitted a linear time trend to estimate marital rates of firearm deaths within each stratum for years 1989 through 2019. This was done separately for firearm suicides and all suicides. We then used the estimated probabilities of being married to randomly apportion deaths with unknown marital status into marital status categories such that the resulting proportion of deaths with a married status matched predicted rates within rounding error of one significant digit.

©2024 Morral AR et al. *JAMA Network Open.*

eAppendix 2. Methodological Details

Overview

We wish to estimate firearm ownership rates for 29 years (1990-2018) for 744 analytic strata defined by the cross classification of 50 states and four dichotomized demographic features (gender, race, marital status, and urbanicity), and then to apply these rate estimates to population estimates for each year and stratum. We use a Bayesian machine learning algorithm, Bayesian Additive Regression Trees (BART), to predict self-reports of firearm ownership found in national surveys as a function of time and strata membership indicators.[4] We use the posterior distribution of predicted rates from this model to estimate ownership rates and their uncertainty for each population stratum in each year. Weighting these strata-level posterior distributions by population sizes allows us to characterize firearm ownership in the United States and over the study period for any aggregation of strata (e.g., at the national, state, or substate levels).[3,5]

Modeling firearm ownership using Bayesian Additive Regression Trees

BART is a flexible Bayesian nonparametric machine learning (ML) algorithm that provides accurate predictions while requiring a minimal set of researcher modeling decisions.[4,13,14] The BART algorithm is broadly similar to other ML approaches using an additive ensemble of regression trees similar to "boosted" models [15] but uses regularizing hyperpriors to control shrinkage and avoid overfit. Unlike many other ML ensemble of trees models, BART optimizes the interaction depth without user input, and simultaneously determines optimal shrinkage/regularization. It can include interactions to the full depth allowed by the number of predictors in our model. The model is estimated using Bayesian (MCMC) methods and produces a posterior distribution of predicted probabilities of firearm ownership, rather than a single point estimate. See Chipman, George, and McCulloch [4] for further discussion of BART models, the theory behind them, and their predictive accuracy relative to other regression algorithms in several

©2024 Morral AR et al. *JAMA Network Open.*

datasets. See Bisbee for a discussion and demonstration of the advantages of a BART model for use in producing longitudinal MRP estimates.[5]

We used BART package (version 2.9), in the R statistical program to estimate the model. The model used a probit link function and error parameterization, which is the BART default for dichotomous outcomes such as household firearm ownership. We used 5-fold cross validation to investigate BART tuning parameters, and to determine whether to include as a predictor strata-level estimated FSS. The final model runs used the BART package defaults for all tuning parameters except the number of trees, which were increased to 200 to reduce CV error. Including strata-level estimates of FSS, discussed in the next section, also slightly improved model CV error. The BART model predicted firearm ownership as a nonlinear function of: the four dichotomous demographic indicators, state, year, survey (BRFSS vs GSS), the strata-level estimate of FSS, and all interactions of these predictors.

Due to relatively high serial correlations across MCMCs we ran the BART model for a large number of samples/iterations and then thinned the posterior to a manageable size. Specifically, we 50,000 iterations after burn-in which were thinned by retaining every 10th, or 5,000.

Consistent with standard practice for MRP estimates (e.g., [16] [17] [18]) the model is estimated on survey data without using standard survey weights. Custom poststratification weights are then applied to the model predictions to produce population representative estimates for populations of interest. This is done for both practical and theoretical reasons. First, neither BART (nor other standard Bayesian models) can include survey weights. Second, hierarchical models have problems when using survey weights where the variables included in the weight derivation are also predictors in the models. Third, by weighting the strata-level estimates to the population, rather than the raw data, one can use far more detailed weights than would be possible without severe variance inflation in a survey with a standard samples size. For these reasons, MRP is sometimes discussed as an alternative to standard survey weighting.[19]

Producing HFR estimates. After estimating the model, we combine strata-specific HFR estimates with population data to produce estimates of HFR for aggregations of strata. We first used the BART model to produce a posterior distribution of the predicted probability of firearm ownership for each of 744 strata in

each year in which a survey was collected. These predictions are conditioned on the full range of geographic and demographic factors included in the model, and the appropriate estimated FSS measure for each strata-year. Predictions are also conditioned on the survey predictor being set as the BRFSS survey. That is, the scale of our HFR measure is tied directly to the specific household firearm ownership question included in BRFSS; the GSS question produces slightly lower rates of ownership than either BRFSS or our HFR measure.

Secondly, we interpolate a posterior distribution of predicted ownership for years in which no survey was given. This was done by estimating ownership for each stratum in the missing year from a random sample of 5000 draws from the combined posterior distributions in years immediately preceding and following the missing year. This was always possible because the GSS was given every two years. While it is possible to get predictions for those years directly out of BART, it does that by systematically using the posterior distribution of an adjacent year. Using our method, in years with no surveys the estimates are agnostic about whether the firearm ownership looks more like the prior or the subsequent year, and the variance of the posterior reflects that uncertainty.

This process creates a matrix of probabilities of firearm ownership for 744 analytic strata across 29 years, each of which is represented as a posterior distribution with over 5000 samples. For each strata-year the population size is then used as a weight, and is applied to each of the posterior samples when producing HFR estimates that combine strata. The reported point estimates are computed as the mean over those 5000 samples, and 95% credible intervals are estimated as the 0.025 and 0.975 percentiles across those samples.

Estimating strata-level FSS

The BART model of firearm ownership includes an estimate of strata-level FSS as a predictor. Here we discuss how these were estimated, why we included them, and the implications of including modeled FSS for use of our HFR estimates.

Construction of the FSS predictors. Instead of using FSS as calculated from raw counts of mortality incidents, we used a model-based measurement approach for estimating strata-level FSS for

©2024 Morral AR et al. *JAMA Network Open.*

inclusion in the HFR model. In contrast to prior studies that evaluate FSS for large aggregates (e.g., states), many of the strata in the current study are small and have no suicides at all over a one- or two-year period. In such cases, direct measures of FSS are either undefined or measured with tremendous variability.[20] To avoid these issues, we use a model-based approach to produce smoothed estimates of strata-level FSS.

Specifically, we modeled suicide mortality data from the Vital Statistics System. The sample was defined by all suicides over the period, and the model predicted the probability that a given suicide was completed with a firearm. Similar to the firearm ownership model, this was modelled using BART, predicting firearm suicide as a function of: state, urbanicity, race, gender and marital status. We then use the model to produce the predicted probability that a suicide would be committed with a firearm for each of the 744 analytic strata in each year. We refer to this model-based measure, which uses partial pooling of information across time, geography and demographic characteristics, as smoothed FSS within the main body of the article to distinguish it from the standard measurement approach. Smoothed FSS is only used as a predictor in our firearm ownership model; when we compare HFR and FSS, we use the standard measures of FSS as we conduct these comparisons only for relatively high-level aggregates of the population. Smoothed FSS values for all years and strata are available online.[21]

Rationale for including smoothed FSS in the model of gun ownership. Including this strata characteristic in the model is done to ensure that the HFR measure of gun ownership retains the same association with suicide outcomes as the survey data on gun ownership on which it is based. If we had not included FSS as a predictor, FSS might have a weaker association with our model-based HFR estimates than it has with the direct survey-based measures of firearm ownership. This bias could occur because all model-based estimates (including those from MRP) partition the total survey variance into a regular component, which is used to construct estimates, and a random, residual component which is ignored in estimates. If those residuals have a systematic association with contextual constructs of interest, like firearm suicide, the model-based estimate may not fully capture the true association between those constructs and the underlying survey data. For this reason, studies doing MRP estimation, or regularized regression models more generally, commonly include characteristics of analytic strata as predictors in their models, even when those strata are also included.[22-24]

©2024 Morral AR et al. *JAMA Network Open.*

It may not be obvious why and how these strata characteristics are being used within a model that also includes strata indicators themselves as predictors. Without regularization, an ensemble of trees model would exactly replicate the sample means for each stratum in the data; in that model the strata-level FSS coefficient would not be identified and thus FSS could not be associated with model residuals. However, regularization, or partial-pooling, almost always improves out-of-sample predictive accuracy, and thus the accuracy of model-based estimates. Including strata-level FSS as a predictor, in addition to the strata indicators themselves, provides another dimension over which partial pooling can occur. This approach does not treat FSS as a proxy measure for firearm ownership and does not assume that the relationship between FSS and ownership is linear, constant over time, or similar across demographic or geographic subgroups. Including this variable only ensures that the regularization within the model does not cause our HFR estimates to misrepresent any true association between FSS and the underlying individual-level gun ownership responses from the surveys.

Using FSS in this way is not treating FSS as a proxy for gun ownership. FSS, because it is constructed with information about suicide rates, is a problematic proxy measure of ownership in research examining suicide as an outcome. In particular, there is a risk that because suicide appears on both the left and the right side of such models, effect estimates may be biased.[25] Essentially, FSS is related to suicide rate outcomes for reasons other than gun ownership, and its effects in a model of suicide outcomes may not accurately or effectively control for gun ownership.

In the context of our HFR model, one can ask if by including FSS in model of survey responses, we are introducing associations between HFR and FSS that are stronger or different than the association of individual ownership and FSS. As discussed above, inclusion of FSS ensures that the observed association between survey responses and FSS is maintained in the HFR estimates. That is, it does not introduce a stronger association between HFR and FSS than found between ownership and FSS, rather it ensures that same association is maintained in the HFR measure of ownership.

©2024 Morral AR et al. *JAMA Network Open.*

eAppendix 3. Supplemental Analyses

Model fit

Model fit was assessed with Tjur's Coefficient of Discrimination, also called Tjur's D.[26] This is a pseudo-R^2 for use with dichotomous outcomes. It is interpretable as the difference between the average model-predicted probability of ownership among survey respondents owning firearms and the average model-predicted probability of ownership among respondents who did not own a firearm. Tjur's D magnitudes are not directly comparable to standard R^2 values from linear models.

For the final model including smoothed FSS as a predictor, respondents who owned a firearm had an average predicted ownership probability of 0.48, while those so did not own a firearm had an average predicted probability of 0.32, giving a Tjur's D of 0.16. This is a small improvement over a similar model without smoothed FSS as a predictor (D = 0.14).

National and state trends in HFR

eFigure 1 illustrates national and state trends in HFR, 1990-2018. eFigure 2 displays national trends in HFR over the same period for each of the 16 demographic strata.

Decomposition of state differences in HFR into portions resulting from demographic vs other differences

To understand how much of state differences in HFR can be explained by their different demographic compositions, we compared our estimated 2018 state HFR rates, to predicted HFR rates in the same year under the assumption that each state had a demographic composition identical to that of the U.S. in 2018. We then calculated the difference between these unadjusted and adjusted state HFR rates and national HFR rates in 2018. As seen in eFigure 3, adjusted HFR rates differ with national rates far less than unadjusted rates for most states, though this is particularly true for states with especially

©2024 Morral AR et al. *JAMA Network Open.*

high HFR rates. This suggests that a large fraction of their higher than national HFR is associated with having a population that is disproportionately comprised of demographic subgroups that have high HFR rates. Across all states, adjusted HFR differed with national HFR by just 57 percent of the differences observed between unadjusted HFR and national rates. That is, demographic composition can explain 43 percent of average state HFR differences with national HFR.

Differences in ownership rates as estimated by the FSS proxy and HFR

As discussed in the main report, we constructed a linear transformation of FSS that provided a least squares best fit for the association between FSS and HFR across all states and years. eFigure 4 illustrates the differences between the FSS proxy and HFR across states. Where these differences are greater than zero, the FSS proxy overestimates ownership. Where they are less than zero, FSS proxy underestimates ownership. In South Dakota, for instance, ownership is underestimated by the FSS proxy by approximately 20 percentage points.

eFigure 5 displays the difference between the FSS proxy and HFR across demographic strata at the national level. Clear time trends in the relationship between the FSS proxy and HFR are evident in this figure.

Detailed state HFR and smoothed FSS findings

eFigure 6 provides HFR and smoothed FSS estimates by year, state, and strata with 95% credible intervals. These are the lowest level estimates in the model, and therefore display estimated rates before any post-stratification is applied (post-stratification is only required when combining strata to construct estimates for aggregated units, like states).

Comparison of HFR and independent estimates of household ownership rates

Although questions on firearm ownership first introduced in 2001 were removed from the core BRFSS questionnaire module after 2004, CDC offers an optional Firearm Safety Module that states can

©2024 Morral AR et al. *JAMA Network Open.*

incorporate into their BRFSS data collection. In addition, in recent years some states have included a set of validated state added questions (SAQs) that cover firearm ownership and safe storage behaviors in their BRFSS collection efforts. Few such state-specific surveys are publicly available, and none were used in the construction of our HFR estimates. Several studies have gained access to these data and published estimated rates of individuals living in a household with a firearm. These published estimates can be used to assess the validity of some of our state and substate HFR estimates. In eTable 1 we compare our HFR estimates to published estimates of firearm ownership based on recent state-specific implementations of the optional BRFSS modules or SAQs.

In most studies we identified, estimates were not provided in a way that was directly comparable with our results. Morgan et al. (2018), for example, provides mean estimates and 95% confidence intervals for overall household firearm ownership in Washington State, but they do not present comparable information by demographic subgroups. However, by applying Bayes' rule to the weighted percentages provided in their Table 1, we were able to calculate an implied mean estimate by demographic subgroups (although not an associated confidence interval). For instance, they indicate that 65.7% of households have no guns, p(nonfirearm)=.657; 55.7% of non-firearm owning households included women, p(female | nonfirearm)=.557; and that 51.6% of all households included women, p(female)=.516. Therefore:

p(firearm | female) = 1-p(nonfirearm | female)

= 1- [p(female | nonfirearm)*p(nonfirearm) / p(female)], by Bayes rule,

= 1- [0.557 * 0.657 / 0.516]

= 0.291

We calculated other such estimates in eTable 1 from Morgan et al. (2018) and Paladhi et al. (2021). As indicated in table notes, there are also some differences in how the samples were constructed across studies and how demographic subgroups were defined that may result in slight differences with our estimates.

Even with these differences, our estimates are similar across all comparisons. With two exceptions, mean estimates of firearm ownership from the state-specific BRFSS's all lie within a few percentage points of our corresponding HFR estimate. The small differences observed in eTable 1 may

©2024 Morral AR et al. *JAMA Network Open.*

well be attributable to differences in methods or sample composition. For instance, the estimated prevalence of household firearm ownership in Kansas produced by Paladhi et al. (2021) is several percentage points higher than our own, though in this year the question Kansas used ("Are any firearms now kept in our around your home") did not instruct respondents to disregard BB guns, non-working firearms, and some other types of guns, possibly leading to higher endorsement rates than expected using the original BRFSS questions (see eAppendix 1).

We also identified one study (Kravitz-Wirtz et al., 2020) that presented firearm ownership estimates based on the California Safety and Well-Being Survey. This survey asks firearm ownership questions that differ substantially from those asked in the BRFSS questionnaires. Specifically, the California Safety and Well-Being Survey asks two questions: (1) "do you or does anyone else you live with currently own any type of gun?", and if yes, (2) "do you personally own a gun?" Based on 2,258 respondents, the firearm ownership prevalence estimates for California in 2018 from these questions were, respectively, 25% (95% CI: 22–28) and 14% (95% CI: 13–16). These bracket our HFR estimate for California in 2018, which is 19.1% (95% CI: 17.2–21.9). This question also may include types of "guns" not counted in our BRFSS estimates, like BB guns, paintball guns, and nonworking firearms.

©2024 Morral AR et al. *JAMA Network Open.*

References

1. Smith TW. General Social Surveys, 1972-2018 [machine-readable data file]/Principal Investigator, Smith, Tom W.; Co-Principal Investigators, Michael Davern, Jeremy Freese, and Stephen Morgan; Sponsored by National Science Foundation. *Chicago: NORC*. 2018:7990-7995.
2. Centers for Disease Control and Prevention. *Behavioral Risk Factor Surveillance System Survey Data*. 2000.
3. Park DK, Gelman A, Bafumi J. Bayesian multilevel estimation with poststratification: State-level estimates from national polls. *Political Analysis*. 2004;12(4):375-385.
4. Chipman HA, George EI, McCulloch RE. BART: Bayesian additive regression trees. *Annals of Applied Statistics*. 2010;4(1):266-298.
5. Bisbee J. Barp: Improving mister p using bayesian additive regression trees. *American Political Science Review*. 2019;113(4):1060-1065.
6. National Cancer Institute. Surveillance, Epidemiology, and End Results (SEER) Program Populations (1969-2020) www.seer.cancer.gov/popdata
7. Arias E, Xu J, Curtin S, Bastian B, Tejada-Vera B. Mortality profile of the non-Hispanic American Indian or Alaska Native population, 2019. 2021;
8. Mihoko Doyle J, Kao G. Are racial identities of multiracials stable? Changing self-identification among single and multiple race individuals. *Social psychology quarterly*. 2007;70(4):405-423.
9. Ruggles S, Flood S, Sobek M, et al. Data from: IPUMS USA: Version 13.0 [dataset]. 2023. *Minneapolis, MN*.
10. Modification Made in 2006 ACS Weighting Methodology-Family Equalization (U.S. Census Bureau) (2007).
11. U.S. Census Bureau. Comparing 2006 American Community Survey Data. https://www.census.gov/programs-surveys/acs/guidance/comparing-acs-data/2006.html
12. Missouri Census Data Center. GeoCore: Geographic correspondence engines. https://mcdc.missouri.edu/applications/geocorr.html
13. Hill JL. Bayesian Nonparametric Modeling for Causal Inference. *Journal of Computational and Graphical Statistics*. 2011;20(1):217-240. doi:10.1198/jcgs.2010.08162
14. Sparapani R, Spanbauer C, McCulloch R. Nonparametric machine learning and efficient computation with Bayesian additive regression trees: the BART R package. *Journal of Statistical Software*. 2021;97:1-66.
15. Friedman JH. Greedy function approximation: a gradient boosting machine. *Annals of statistics*. 2001:1189-1232.
16. Gelman A. Poststratification into many categories using hierarchical logistic regression. *Survey methodology*. 1997;23:127.
17. Ghitza Y, Gelman A. Deep interactions with MRP: Election turnout and voting patterns among small electoral subgroups. *American Journal of Political Science*. 2013;57(3):762-776.
18. Lax JR, Phillips JH. How should we estimate public opinion in the states? *American Journal of Political Science*. 2009;53(1):107-121.
19. Buttice MK, Highton B. How does multilevel regression and poststratification perform with conventional national surveys? *Political analysis*. 2013;21(4)
20. Kang MS, Rasich EA. Extending the Firearm Suicide Proxy for Household Gun Ownership. *Am J Prev Med*. May 7 2023;
21. Morral AR, Smart R, Schell TL, Vegetabile B, Thomas E. Interactive visualization of geographic differences in the proportion of individuals living in a household with a firearm. RAND Corporation. 2024. https://rand.shinyapps.io/hfa-estimates/
22. Broniecki P, Leemann L, Wüest R. Improved Multilevel Regression with Poststratification through Machine Learning (autoMrP). *The Journal of Politics*. 2022;84(1):597-601.

©2024 Morral AR et al. *JAMA Network Open.*

23. Hanretty C, Lauderdale BE, Vivyan N. Comparing strategies for estimating constituency opinion from national survey samples. *Political Science Research and Methods*. 2018;6(3):571-591.
24. Warshaw C, Rodden J. How should we measure district-level public opinion on individual issues? *The Journal of Politics*. 2012;74(1):203-219.
25. National Research Council. *Firearms and Violence: A Critical Review*. National Academies of Science; 2004.
26. Tjur T. Coefficients of determination in logistic regression models—A new proposal: The coefficient of discrimination. *The American Statistician*. 2009;63(4):366-372.

©2024 Morral AR et al. *JAMA Network Open.*

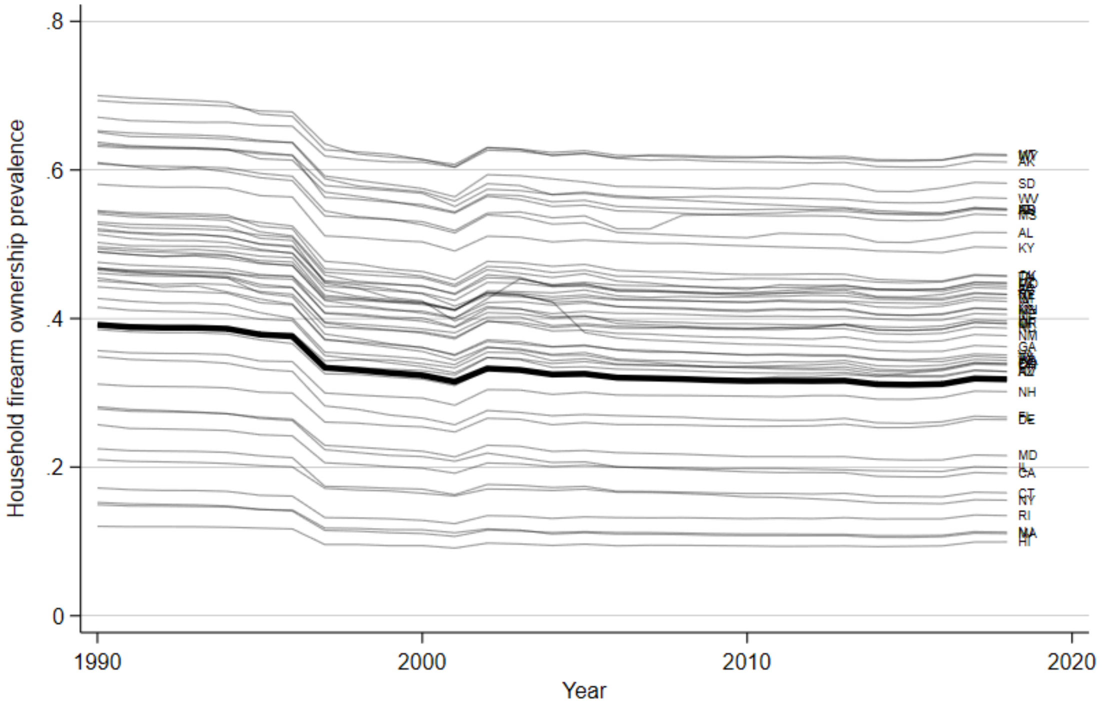

eFigure 1. Change in Household Firearm Ownership Rate, National (Thick Black Line) and by State, 1990-2018

©2024 Morral AR et al. *JAMA Network Open.*

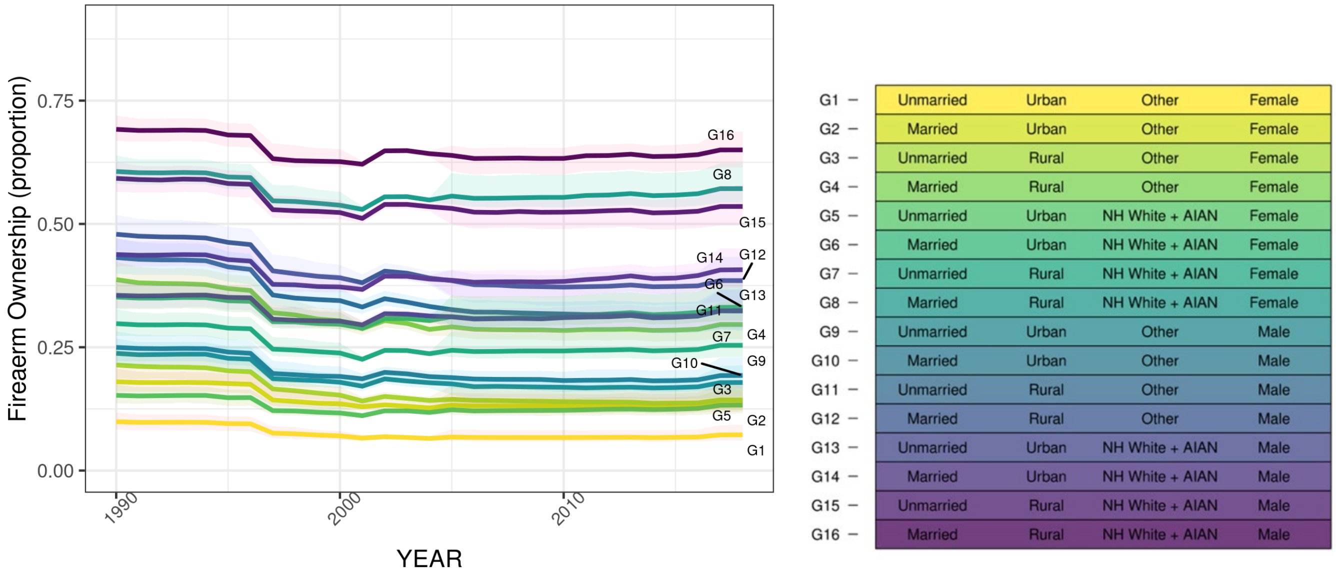

eFigure 2. Estimates of the proportion of individuals living in households with firearms for 16 U.S. demographic subgroups (with 95% credible intervals).

©2024 Morral AR et al. *JAMA Network Open.*

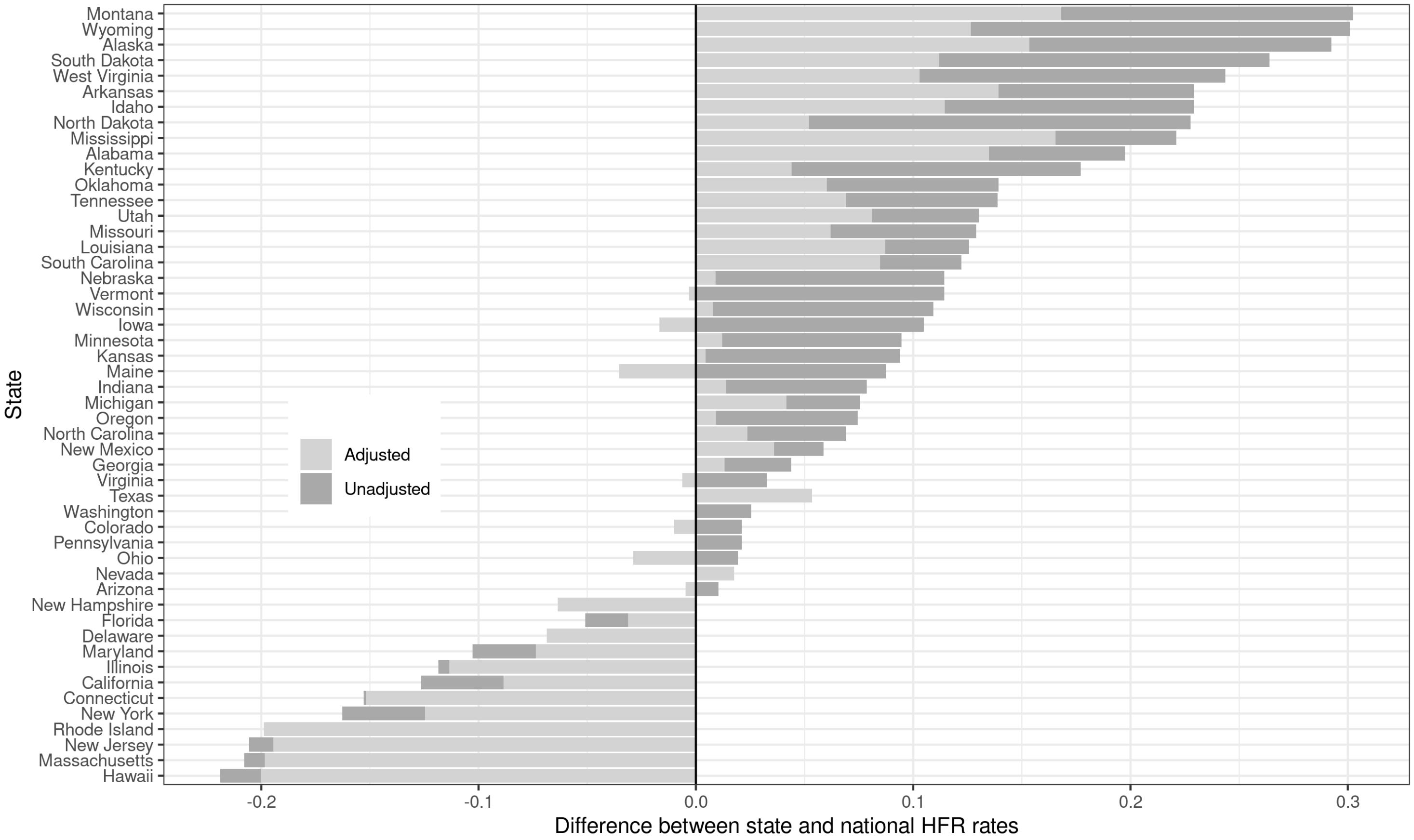

eFigure 3 Differences between state and national HFR rates in 2018: total difference (unadjusted) and after adjusting for demographic differences between states and the nation.

©2024 Morral AR et al. *JAMA Network Open.*

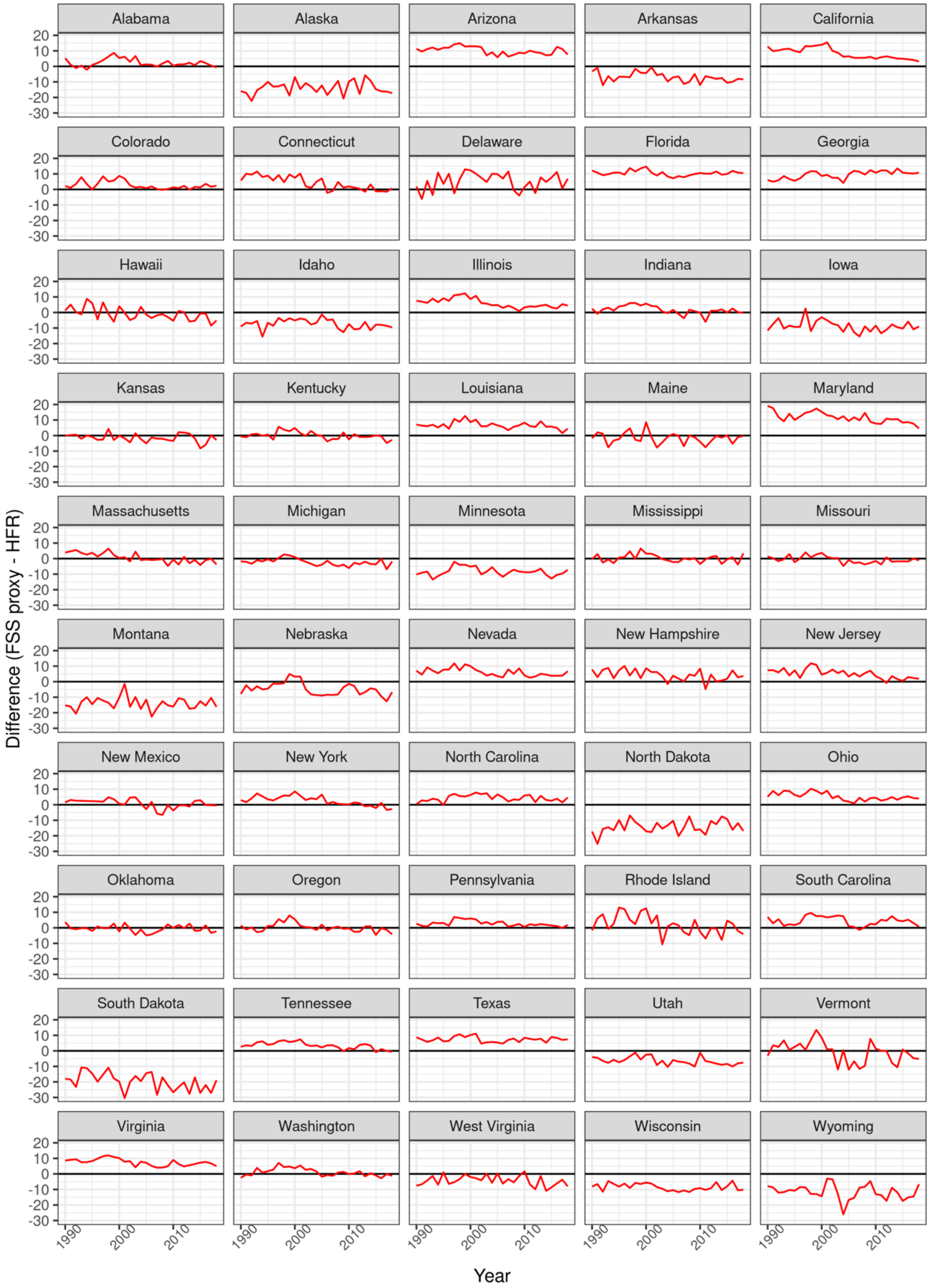

eFigure 4. Differences between firearm ownership rates as proxied by FSS and survey-based HFR estimates, by state and year

©2024 Morral AR et al. *JAMA Network Open.*

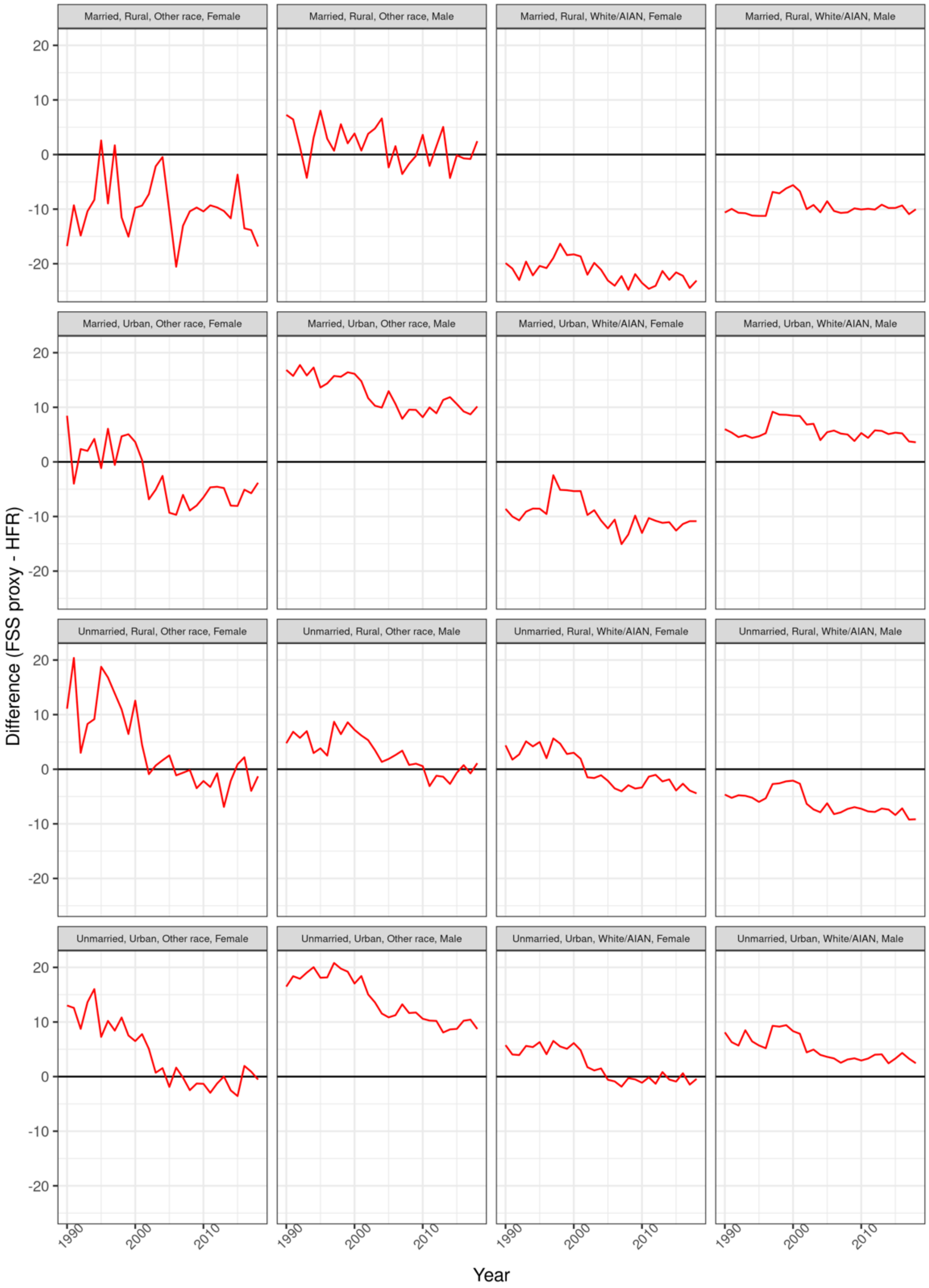

eFigure 5. Differences between firearm ownership rates as proxied by FSS and survey-based HFR estimates, by subgroup and year

©2024 Morral AR et al. *JAMA Network Open.*

eFigure 6 Legend: Strata labels and colors

G1	Unmarried	Urban	Other	Female
G2	Married	Urban	Other	Female
G3	Unmarried	Rural	Other	Female
G4	Married	Rural	Other	Female
G5	Unmarried	Urban	NH White + AIAN	Female
G6	Married	Urban	NH White + AIAN	Female
G7	Unmarried	Rural	NH White + AIAN	Female
G8	Married	Rural	NH White + AIAN	Female
G9	Unmarried	Urban	Other	Male
G10	Married	Urban	Other	Male
G11	Unmarried	Rural	Other	Male
G12	Married	Rural	Other	Male
G13	Unmarried	Urban	NH White + AIAN	Male
G14	Married	Urban	NH White + AIAN	Male
G15	Unmarried	Rural	NH White + AIAN	Male
G16	Married	Rural	NH White + AIAN	Male

©2024 Morral AR et al. *JAMA Network Open.*

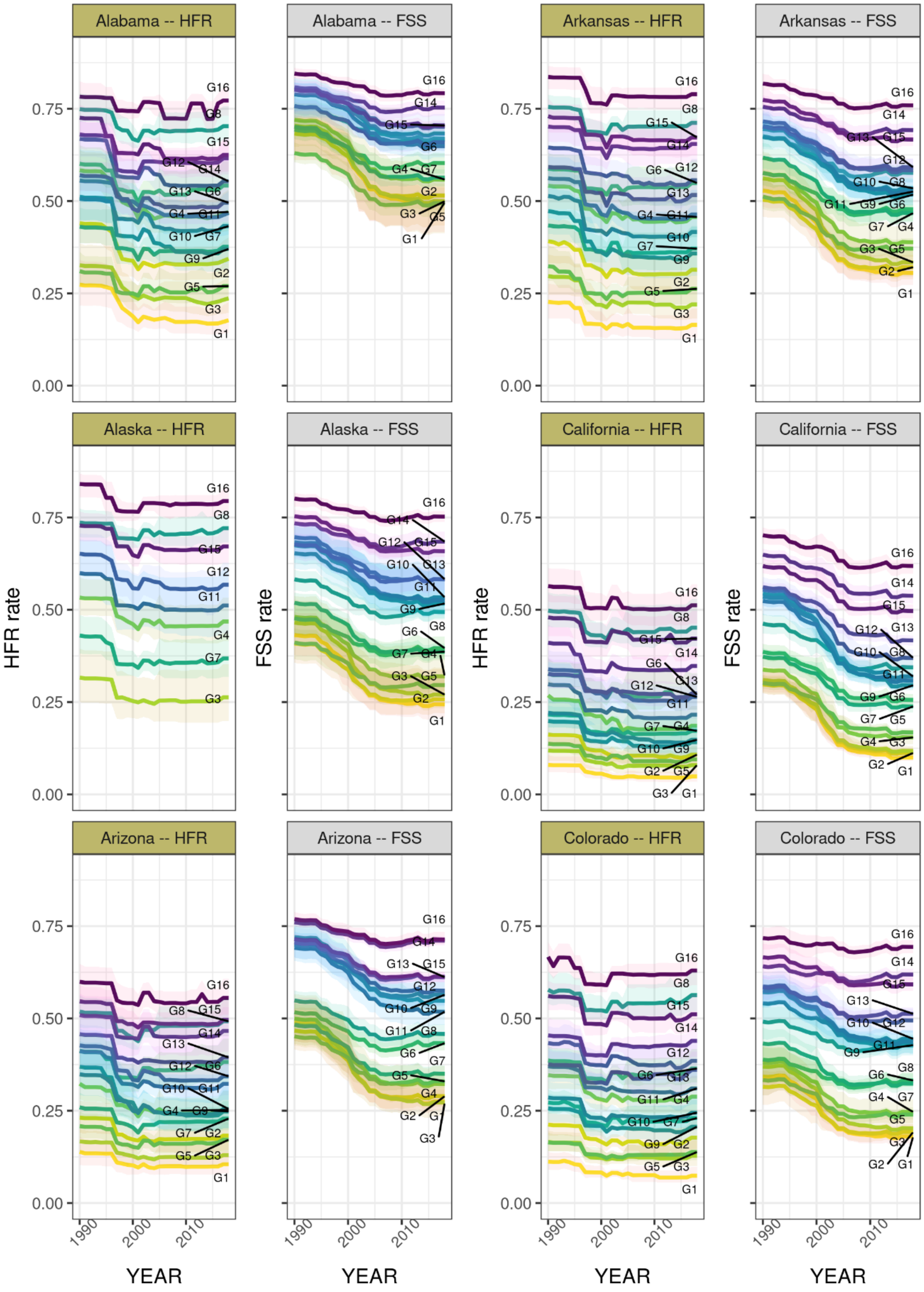

eFigure 6. State household firearm ownership rates (HFR) and FSS rates by demographic subgroups and years

©2024 Morral AR et al. *JAMA Network Open.*

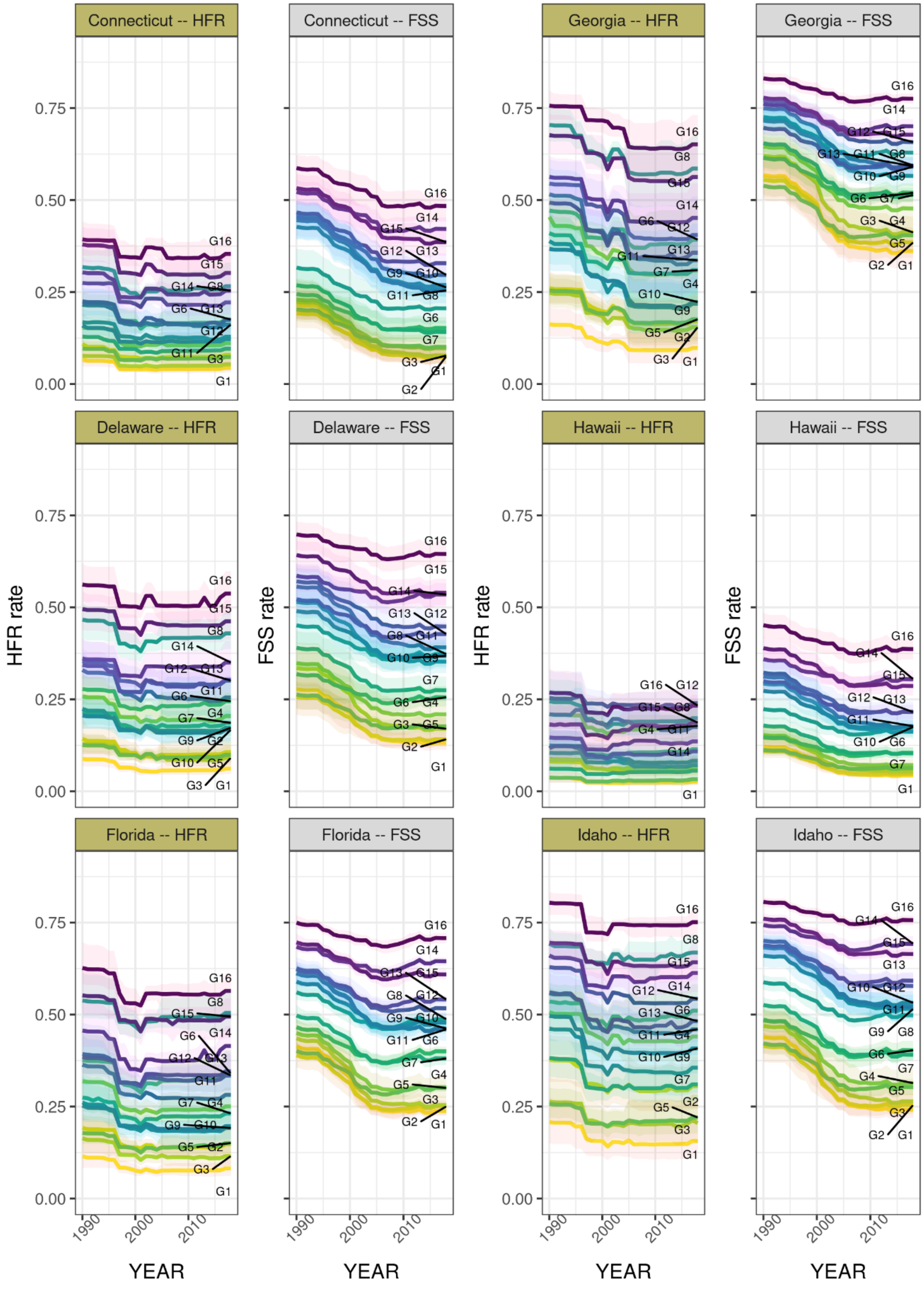

eFigure 6 (cont.) State household firearm ownership rates (HFR) and FSS rates by demographic subgroups and years

©2024 Morral AR et al. *JAMA Network Open.*

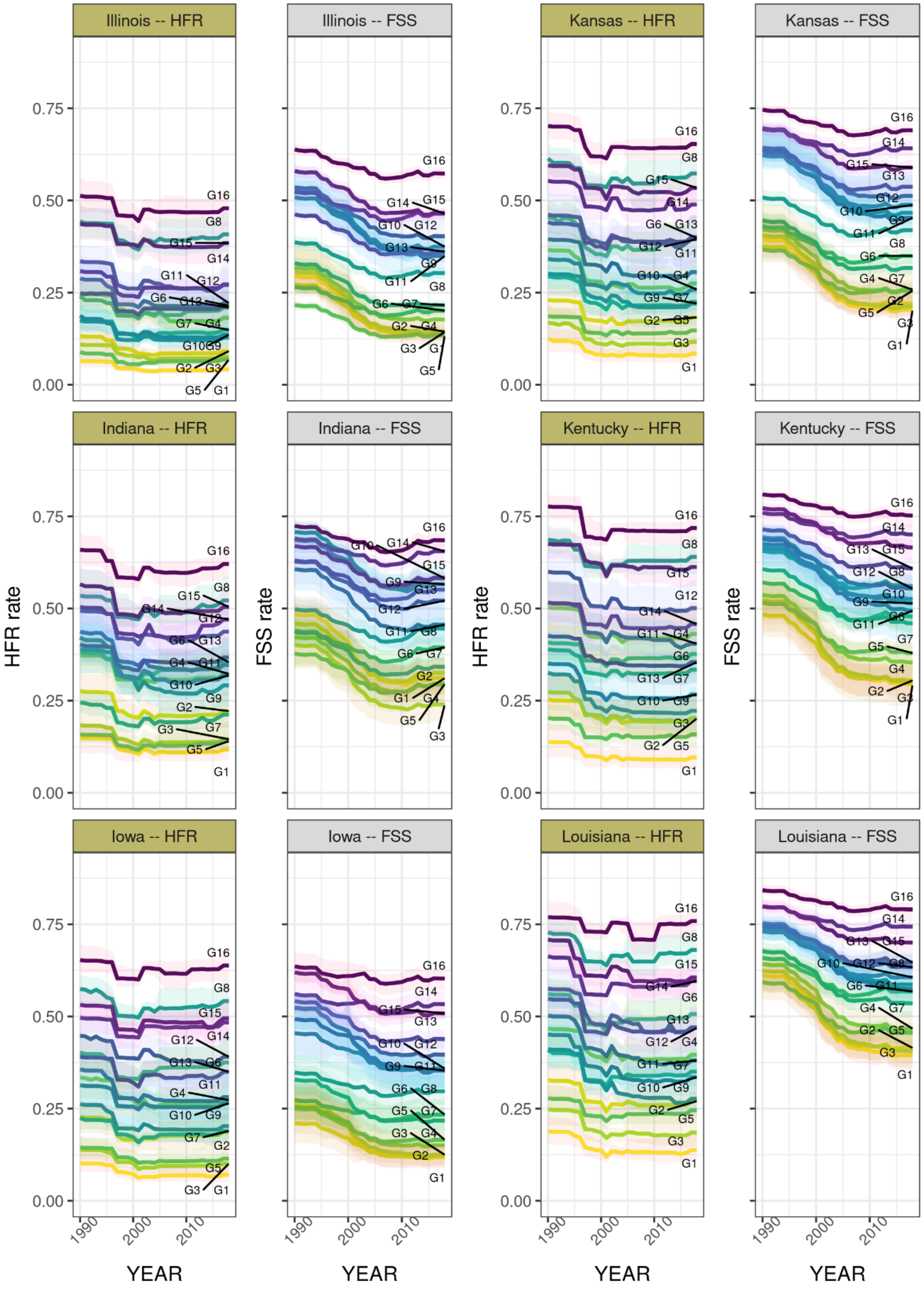

eFigure 6 (cont.) State household firearm ownership rates (HFR) and FSS rates by demographic subgroups and years

©2024 Morral AR et al. *JAMA Network Open.*

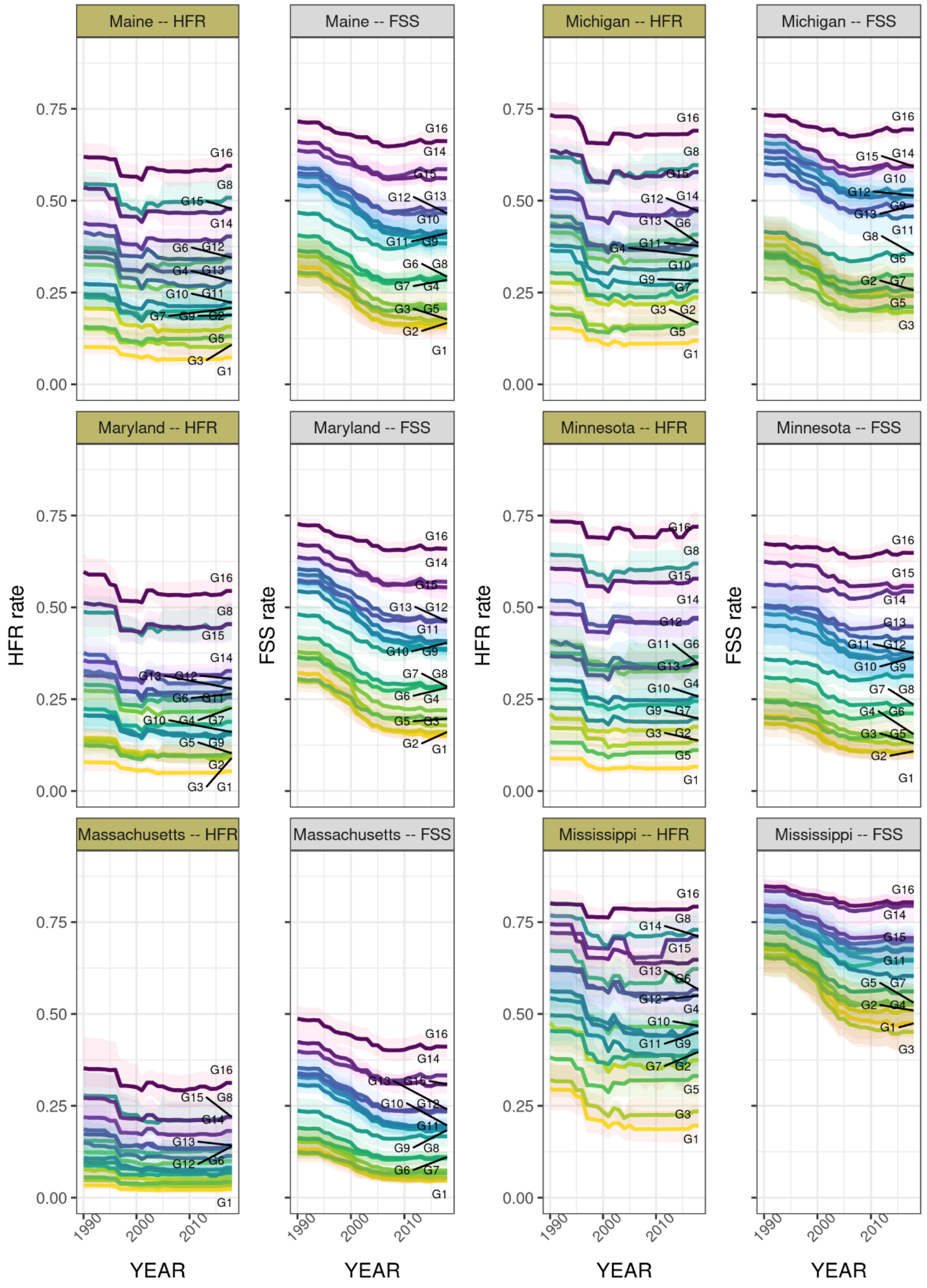

eFigure 6 (cont.) State household firearm ownership rates (HFR) and FSS rates by demographic subgroups and years

©2024 Morral AR et al. *JAMA Network Open.*

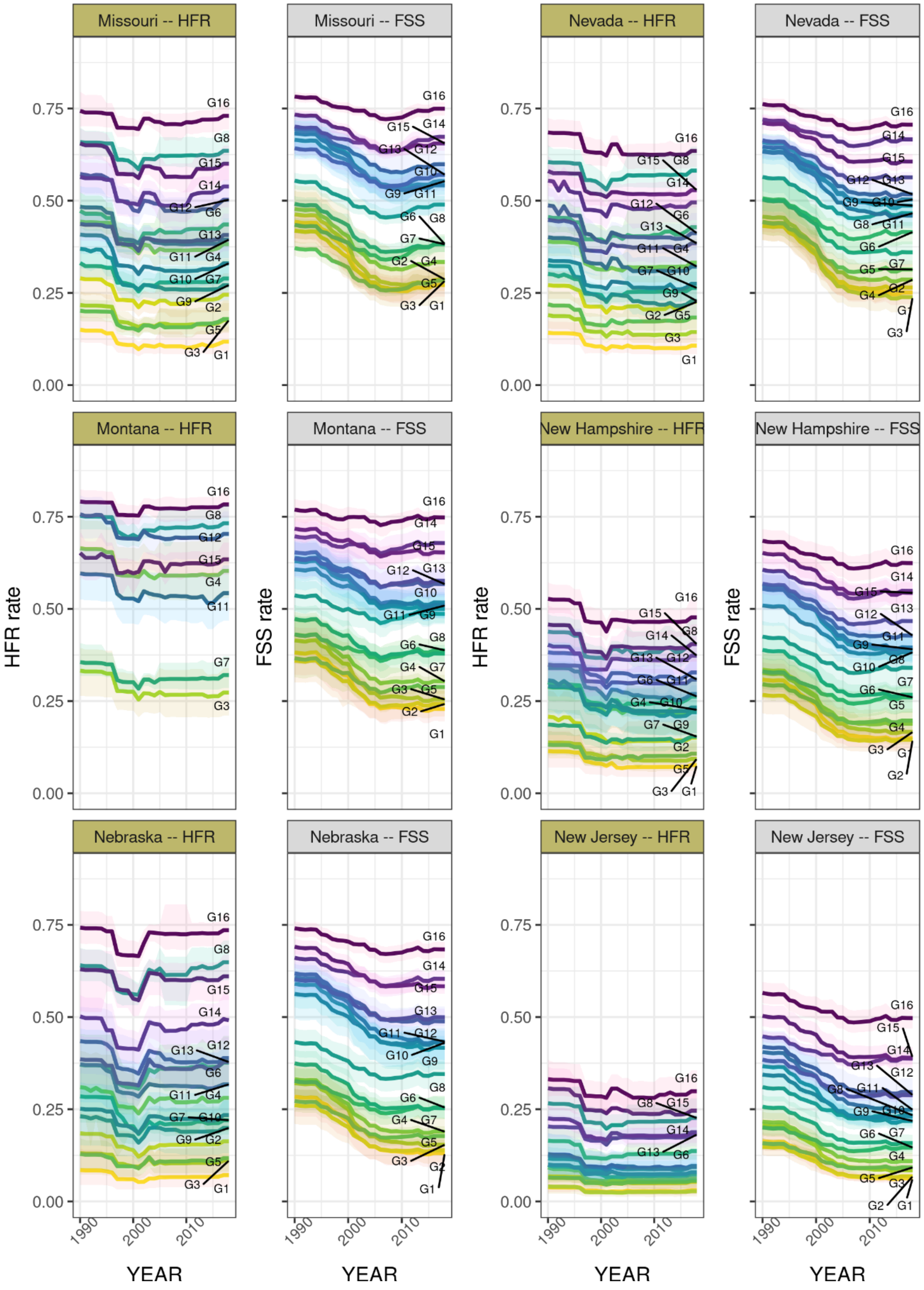

eFigure 6 (cont.) State household firearm ownership rates (HFR) and FSS rates by demographic subgroups and years

©2024 Morral AR et al. *JAMA Network Open.*

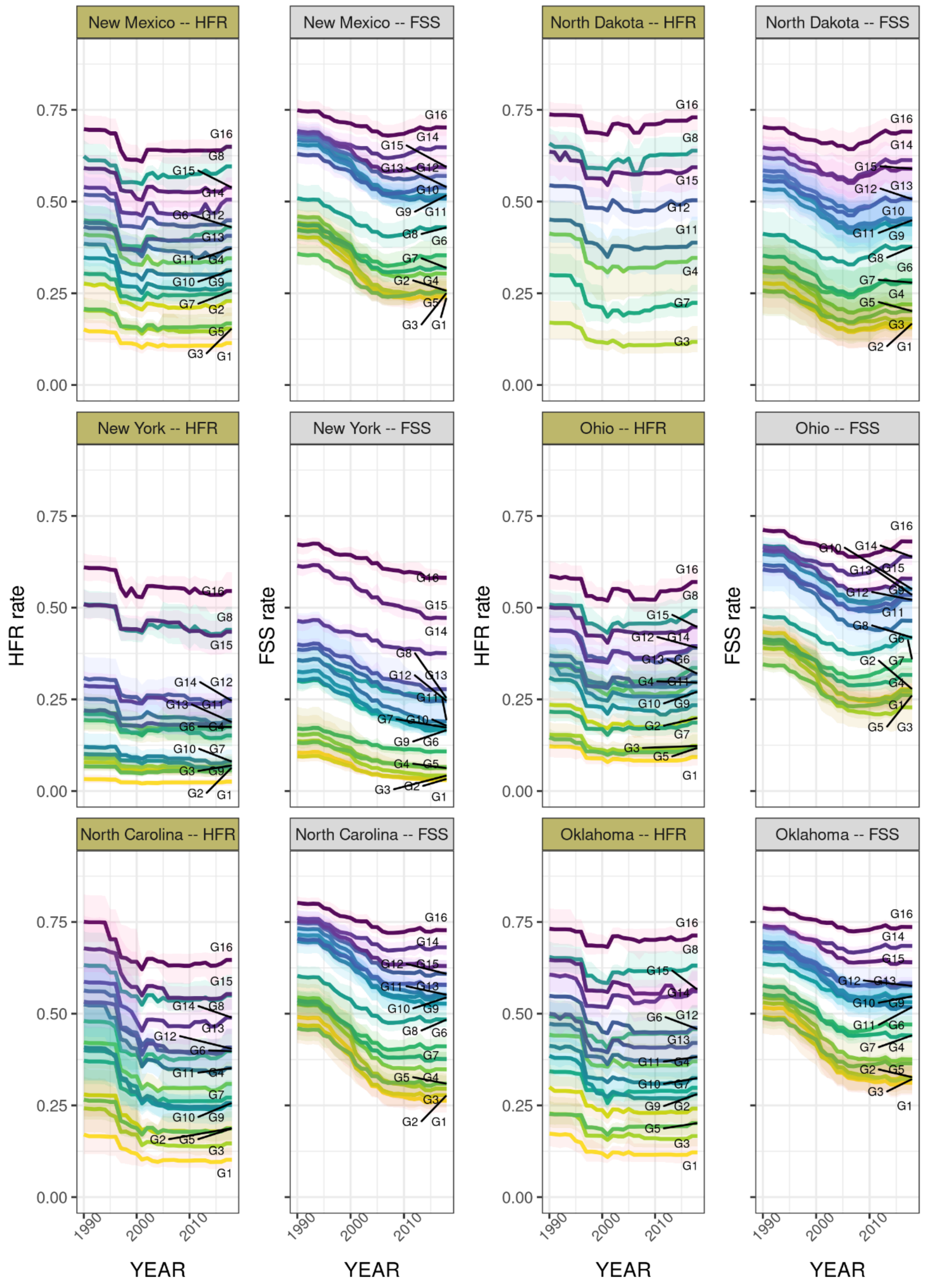

eFigure 6 (cont.) State household firearm ownership rates (HFR) and FSS rates by demographic subgroups and years

©2024 Morral AR et al. *JAMA Network Open.*

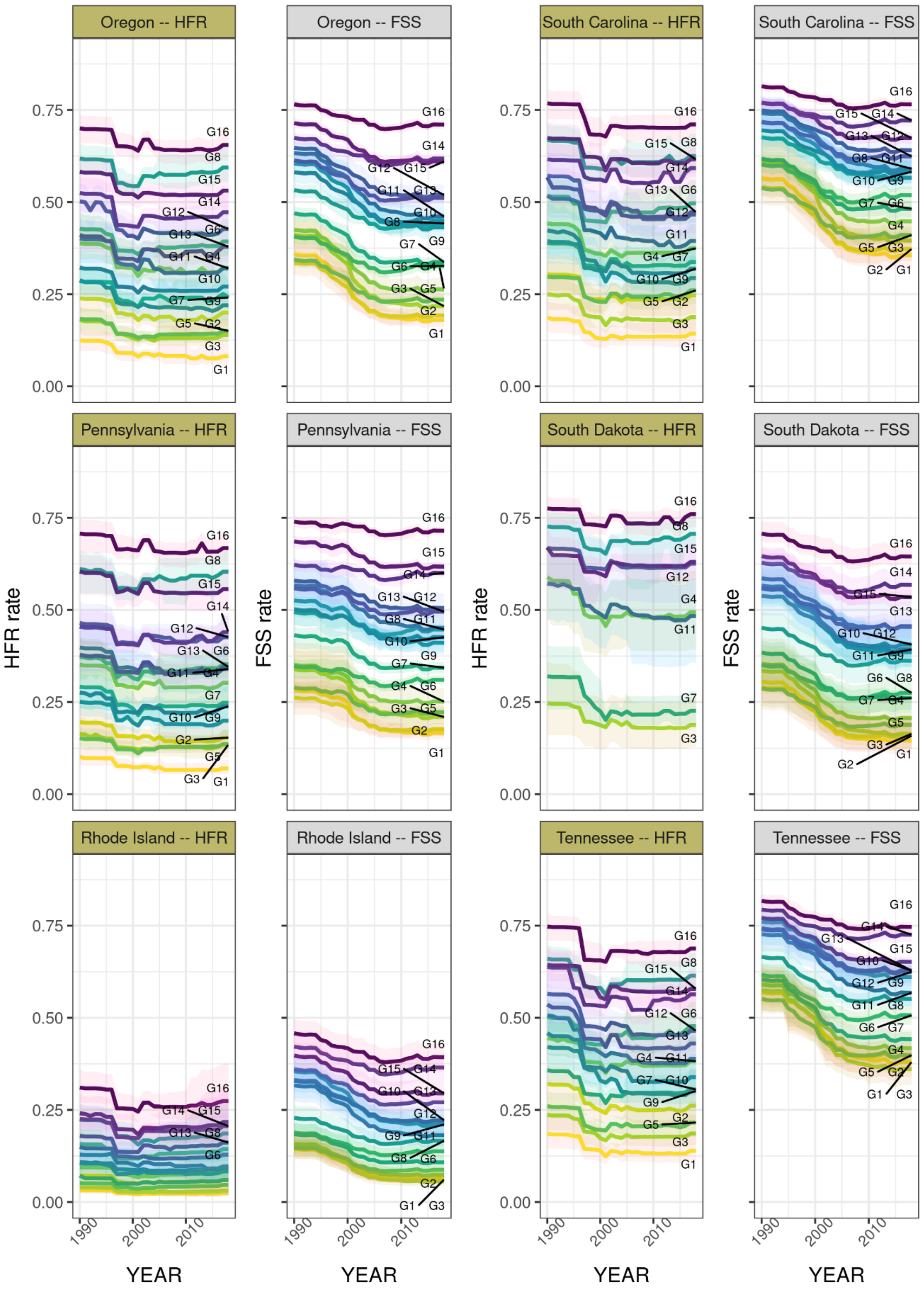

eFigure 6 (cont.) State household firearm ownership rates (HFR) and FSS rates by demographic subgroups and years

©2024 Morral AR et al. *JAMA Network Open.*

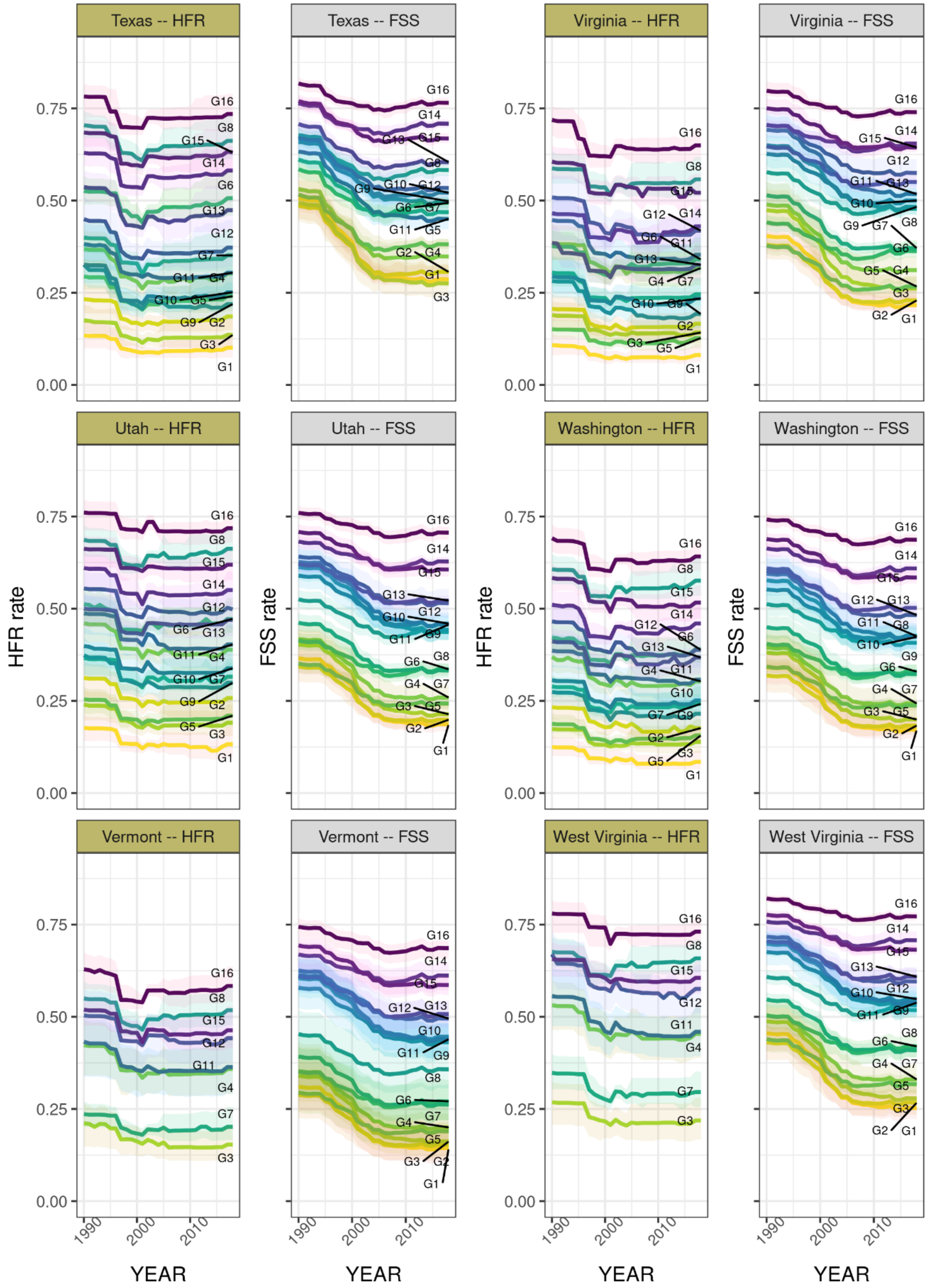

eFigure 6 (cont.) State household firearm ownership rates (HFR) and FSS rates by demographic subgroups and years

©2024 Morral AR et al. *JAMA Network Open.*

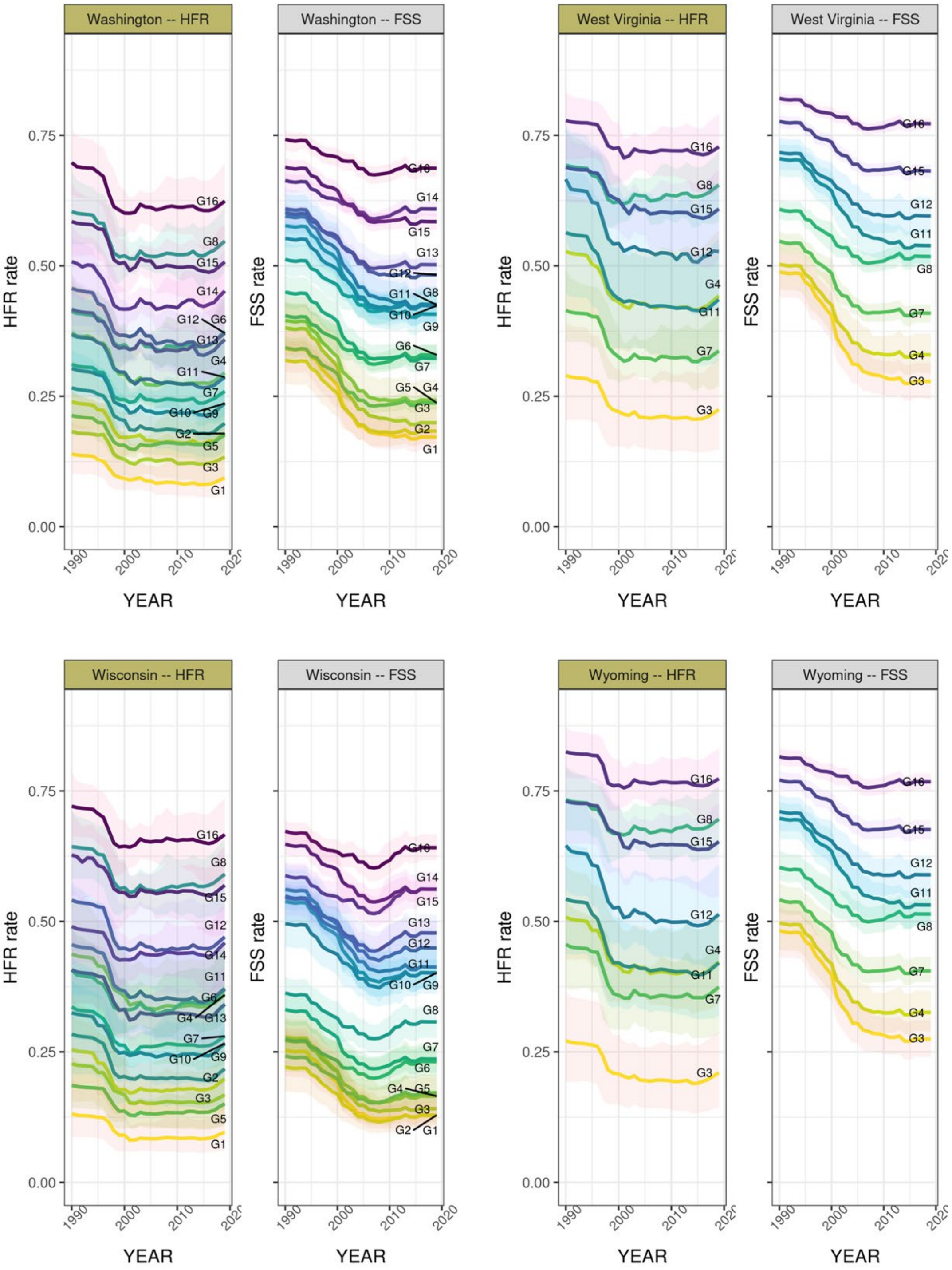

eFigure 6 (cont.) State household firearm ownership rates (HFR) and FSS rates by demographic subgroups and years

©2024 Morral AR et al. *JAMA Network Open.*

eTable. Comparisons of HFR with state and substate-level estimates from state-specific BRFSS module data presented in published literature

Study	Timeframe	State	N respondents	Subgroup	Study estimate (95% CI)	RAND HFR estimate (95% CI)
Morgan et al.	2013, 2015, 2016	Washington	34,884	All	34.3 (33.7–35.0)	33.7 (32.0–36.3)
				Female	29.1	27.1 (25.2–31.0)
				Male	39.9	40.5 (37.3–43.4)
				Non-Hispanic White[b]	39.9	38.0 (36.3–40.6)
				Married	38.5	41.5 (39.5–44.2)
Paladhi et al.[c]	2017	Idaho	3,827	All	57.0	54.8 (52.1–58.5)
		Oregon	3,779	All	40.0	39.4 (36.9–43.7)
		Texas	8,658	All	38.9	34.9 (32.4–39.8)
		California	3,019	All	21.7	19.2 (17.3–22.0)
		Kansas	8,251	All	45.1	41.3 (38.7–45.0)
		Utah	4,354	All	47.0	44.9 (42.2–48.9)
Horn et al.[d]	2017	California	Not stated	All	20.6	19.2 (17.3–22.0)
		Idaho	Not stated	All	55.8	54.8 (52.1–58.5)

Note: All RAND HFR estimates are for 2017, except those for Morgan estimates, which are taken from 2016.

[b]Morgan et al. (2018) do not include AI/AN in this group but our HFR categorization does.

[c]Sample restriction for this study requires participants to answer both the firearm module questions and all four healthcare access modules in the BRFSS survey.

[d]Sample restriction for this study requires participants to answer both the firearm and mental health questions in the BRFSS survey.

©2024 Morral AR et al. *JAMA Network Open.*
