# Supplementary material for: Geographic and Demographic Differences in the Proportion of Individuals Living in Households With a Firearm, 1990-2018
Source: JAMA Netw Open. 2024 Feb 28;7(2):e240562. doi: 10.1001/jamanetworkopen.2024.0562 (PMC10902733; doi:10.1001/jamanetworkopen.2024.0562)
Supplement: Supplement 2. — Data Sharing Statement [file jamanetwopen-e240562-s002.pdf]

Data Sharing Statement

Morral. Geographic and Demographic Differences in the Proportion of Individuals Living in Households With a Firearm, 1990-2018. *JAMA Netw Open*. Published February 28, 2024. doi:10.1001/jamanetworkopen.2024.0562

Data
Data available: Yes
Data types: Data (not involving human participants)
How to access data: https://rand.shinyapps.io/hfa-estimates/
When available: With publication

Supporting Documents
Document types: None

Additional Information
Who can access the data: Publicly available
Types of analyses: For any purpose
Mechanisms of data availability: Without investigator support
Any additional restrictions: Data available with attribution
